# Current Insights on Biomarkers in Lupus Nephritis: A Systematic Review of the Literature

**DOI:** 10.3390/jcm11195759

**Published:** 2022-09-28

**Authors:** Leonardo Palazzo, Julius Lindblom, Chandra Mohan, Ioannis Parodis

**Affiliations:** 1 Division of Rheumatology, Department of Medicine Solna, Karolinska Institutet, 171 77 Stockholm, Sweden; 2 Medical Unit of Gastroenterology, Dermatology and Rheumatology, Karolinska University Hospital, 171 76 Stockholm, Sweden; 3 Department Biomedical Engineering, University of Houston, Houston, TX 77204, USA; 4 Department of Rheumatology, Faculty of Medicine and Health, Örebro University, 701 82 Örebro, Sweden

**Keywords:** systemic lupus erythematosus, lupus nephritis, biomarkers, diagnosis, monitoring, prognosis

## Abstract

Lupus nephritis (LN) is a major cause of morbidity and mortality among patients with systemic lupus erythematosus (SLE). However, promising emerging biomarkers pave the way toward an improved management of patients with LN. We have reviewed the literature over the past decade, and we herein summarise the most relevant biomarkers for diagnosis, monitoring, and prognosis in LN. An initial systematic search of Medline was conducted to identify pertinent articles. A total of 104 studies were selected to be included in this review. Several diagnostic biomarkers, including MCP-1, TWEAK, NGAL, and uric acid, exhibited good ability to differentiate LN patients from non-renal SLE patients. Several cytokines and chemokines, including IL-10, IL-17, MCP-1, and IP-10, hold promise for assessing LN disease activity, as do cell adhesion molecules (CAMs). Angiogenesis-related and haemostasis-related proteins have also displayed potential for monitoring disease activity. Biomarkers of responses to therapy include Axl, CD163, and BAFF, whereas VCAM-1, ALCAM, and ANCAs have been reported as prognostic markers, along with traditional markers. In addition, novel renal tissue biomarkers may prove to be a useful complement to histological evaluations. The overall heterogeneity of the inclusion criteria and outcome measures across different studies, along with a lack of validation in multi-centre cohorts, call for future collaborative efforts. Nevertheless, we foresee that several biomarkers hold promise toward optimisation of the management of LN, with the use of integrated omics and panels of less invasive biomarkers paving the way towards personalised medicine.

## 1. Introduction

Kidney involvement in patients with systemic lupus erythematosus (SLE), termed lupus nephritis (LN), is a common and severe manifestation of the disease. The prevalence of LN varies depending on age, sex, and ethnicity, among other factors, and it is estimated to be 35–60% [1,2]. The clinical presentation is highly heterogneous, varying from silent disease to rapidly progressive nephropathy [3,4]. Despite advancements in the understanding of the pathophysiology of LN, as well as in the development of treatment strategies, only 50–70% of patients achieve remission, and LN is still a major cause of mortality and morbidity in patients with SLE [5].

A kidney biopsy is still the gold standard for the evaluation of LN for the confirmation of LN diagnosis and determination of the type and degree of kidney tissue injury and damage [6,7]. However, a kidney biopsy is an invasive procedure, and its utility in guiding therapeutic decisions is limited by the heterogeneity of the etiopathogenesis of renal disease in patients with SLE [5]. Fluid-based biomarkers, that are validated indicators of physiologic or pathologic processes or responsiveness to therapy [8], constitute promising complemental or alternative, less invasive modes for assessing renal SLE.

Traditional laboratory biomarkers include immune serology tests, such as anti-double-stranded (ds) DNA and complement levels, and kidney disease-related parameters, such as proteinuria estimated through 24-h urinary protein excretion or the urine protein-to-creatinine ratio (uPCR), urinary sediment, and glomerular filtration rate (GFR). These are well-established tools for the clinical evaluation of LN. However, they have shown the unsatisfactory ability to detect LN flare development early, as well as limited sensitivity and specificity to discriminate between active ongoing disease and chronic organ damage, which is crucial for treatment planning [9,10].

Recent technological advancements, including assays for broad proteomic and metabolomic analyses, have contributed to a growing body of evidence on new biomarkers, some of which have shown equal or even superior performances than traditional markers [11,12,13]. This review aims to summarise the literature of the last decade on the topic of biomarkers of potential utility for the diagnosis, monitoring, and prognosis of adult patients with LN.

## 2. Materials and Methods

An initial systematic literature search was performed to identify the relevant articles addressing the potential role of biomarkers in the evaluation of diagnosis, disease activity, organ damage, responsiveness to therapy, and prognosis over the long term in adult patients with LN (≥18 years of age). Details regarding the search are provided in the online Supplementary Materials (Figure S1 and Table S1), and the Preferred Reporting Items for Systematic reviews and Meta-Analyses (PRISMA) 2020 flow diagram presented in Figure S1 synthetises the key points of the selection process [14]. The search terms are detailed in Table S1. The search strategy was applied to the Medline database, and studies in English published between 1 January 2012 and 13 June 2022 were assessed for eligibility. The study designs deemed eligible comprised meta-analyses and randomised controlled trials (RCTs), including post hoc analyses of RCTs, quasi-experimental studies, cohort studies, and cross-sectional studies. Animal studies, preclinical studies, studies of qualitative design, case series, case reports, studies on paediatric LN, and studies focused on comorbidities were beyond the scope of this review. The assessment for eligibility was performed by two investigators (L.P. and J.L.). Ambiguities were discussed with a third investigator (I.P.) to arrive at a consensus.

Ninety-three reports met the inclusion criteria, and fourteen additional articles were selected based on an expert opinion (C.M. and I.P.), yielding a total of one hundred and seven studies included in this systematic review. To evaluate the risk of bias (RoB) within the included studies, the Joanna Briggs Institute (JBI) Critical Appraisal Checklist for Analytical Cross-Sectional Studies, the JBI Critical Appraisal Checklist for Systematic Reviews and Research Syntheses, and the JBI Critical Appraisal Checklist for Cohort Studies [15] were used (Supplementary Tables S2–S4). The ethnic characteristics of study populations for each one of the studies included in the review are provided in Supplementary Table S5.

## 3. Results

### 3.1. Diagnostic Biomarkers

Autoantibodies and immune complexes are hallmarks of SLE, hypothesised to be directly involved in the pathogenesis underlying organ injury in SLE, including LN [16]. Anti-dsDNA and anti-C1q constitute well-known autoantibodies that are widely used in clinical practice and recommended by guidelines as surveillance tools [6]. Additionally, new autoantibodies are emerging, among which anti-α-enolase (anti-ENO-1) antibodies deserve mention. Anti-ENO-1 form as a consequence of α-enolase externalisation during NETosis [17]. In a study by Huang et al., anti-ENO-1 displayed a good ability to predict the incidence of LN in SLE patients (area under the curve (AUC)= 0.81; *p* = 0.001) [18]. Interestingly, studies conducted by Bruschi et al. revealed a higher prevalence of the anti-ENO-1 IgG2 isotype in LN patients compared with subjects with other glomerulonephritides, as well as in patients with LN, compared with patients with non-renal SLE (AUC = 0.82; *p* = 0.02) [19,20,21].

Hyperuricemia, a marker of renal disfunction, has recently been hypothesised to be involved in LN pathogenesis [22]. Consistent results across different studies lend support to a diagnostic role of uric acid (UA) for distinguishing LN from SLE with no renal involvement (AUC = 0.80–0.86; *p* < 0.001 for all; sensitivity (sens.): 75–83%; specificity (spec.): 70–79%; positive predictive value (PPV): 70–87%; negative predictive value (NPV): 62–80%; cut-off values: 4.47–5.54 mg/dL) [22,23,24].

Tumour necrosis factor (TNF) weak inducer of apoptosis (TWEAK) is a cytokine member of the TNF superfamily involved in multiple cellular processes [25]. It is postulated that the main source of TWEAK in LN are innate immune cells, mainly monocytes and natural killer cells, which infiltrate the kidneys during inflammation. TWEAK exerts its action by interacting with its sole receptor, Fn14, and activates multiple intracellular pathways, which vary depending on the cell microenvironment [26,27]. TWEAK has been linked to LN pathogenesis and represents a possible target of renoprotective agents [28,29]. Pathological effects mediated by TWEAK/Fn14 signalling include the enhancement of apoptosis of parenchymal renal cells and induction of proinflammatory cytokines and chemokines, which, in turn, might serve as emerging biomarkers of kidney injury in LN [27,30]. Both serum and urinary TWEAK hold promise as useful diagnostic markers of LN, with the latter displaying outstanding overall metrics in most studies (see Table 1 for the detailed metrics) [26,27,30], although two studies reported the superiority of serum TWEAK over urinary TWEAK in the ability to differentiate between LN and non-renal SLE [31,32].

Neutrophil gelatinase-associated lipocalin (NGAL) is a small secreted glycoprotein whose expression is upregulated in several pathologic conditions, including inflammatory and renal diseases [33]. NGAL is functionally involved in the induction of apoptosis and iron sequestration, thus playing a critical role in innate immunity in the context of bacterial infections [34]. With regard to LN, NGAL is highly expressed by activated leukocytes and tubular epithelial cells in response to inflammation and kidney injury [35]. NGAL may act as a nephroprotective agent by modulating apoptosis in resident macrophages and tubular cells in the context of a renal insult, although a definitive mechanistic explanation for its function in LN has yet to be provided [34]. Urinary NGAL has proven to be a versatile biomarker in LN across multiple studies [35,36,37,38,39,40,41]. Of relevance to the diagnosis of LN, the urine levels of NGAL were found to be higher in patients with LN than in patients with non-renal SLE in two independent cohorts, exhibiting an overall good diagnostic ability (AUC = 0.70–0.99; sens.: 67–98%; spec.: 63–100%) [41,42].

A growing number of studies have investigated the role of microRNAs (miRNAs) as putative epigenetic biomarkers in LN. These short single-stranded RNA molecules have been implicated in multiple regulatory events of gene expression [43], and differential levels of different miRNAs, including miRNA-21, miRNA-146a, miRNA-150, miRNA-155, miRNA-181a, miRNA-223, and miRNA-423, have been linked to kidney involvement in patients with SLE [44,45,46,47]. In particular miRNA-21 displayed a satisfactory ability to distinguish between patients with active LN compared with a group of patients with either inactive LN or non-renal SLE (AUC = 0.89; *p* < 0.001) [45], as well as patients with LN from healthy controls (AUC = 0.91; sens.: 86%; spec.: 63%; PPV: 76%; NPV: 93%; *p* < 0.001) [46].

Recently, monocyte-derived urinary microparticles (MPs) have been shown to be promising diagnostic biomarkers for LN, serving as a remote biopsy to assess the retainment of inflammatory cells within renal parenchyma [48]. Burbano et al. found a significant increase of MP-HLADR^+^, MP-high mobility group box 1 (HMGB1)^+^, and MP-C-X3-C chemokine receptor 1 (CX3CR1)^+^ in patients with LN compared to patients with non-renal SLE (see Table 1 for the metrics). In addition, MP-HMGB1^+^ exhibited an adequate performance in differentiating patients with active LN from patients with inactive LN (AUC = 0.83; sens.: 55%; spec.: 93%; *p* < 0.001) [48]. Interestingly, the aforementioned molecules are mainly expressed by non-classic monocytes, lending support to the hypothesis that this specific cell subset constitutes one of the main drivers of kidney inflammation and injury in LN [48,49].

A summary of the diagnostic biomarkers of LN as derived from the systematic search of the literature conducted herein is presented in Table 1.

### 3.2. Biomarkers of Disease Activity and Organ Damage

Cytokines and chemokines play a critical role in the development of LN, e.g., in the process of recruiting leukocytes and orchestrating the inflammatory response [12,76]. Serum IL-10 and IL-17 have been shown to exhibit satisfactory performances in discriminating between patients with active LN and patients with inactive LN and displayed a strong correlation with the disease activity parameters (see Table 2 for the detailed metrics) [61,77]. Several studies have investigated the potential of cytokines and chemokines as urinary biomarkers [11,61,78,79,80]. Using an innovative electrochemiluminescence-based multiplex panel, Stanley et al. identified and validated five selected urinary proteins (IL-7, IL-12p40, IL-15, thymus- and activation-regulated chemokine (TARC), and interferon-γ (IFN-γ) inducible protein-10/C-X-C Motif Chemokine Ligand 10 (IP-10/CXCL10)), which displayed diagnostic potential and strong correlations with the renal domain of Systemic Lupus Erythematosus Disease Activity Index (rSLEDAI) (r = 0.67–0.74; *p* < 0.001 for all) [78]. Of these, IP-10 was also investigated in other studies, which overall corroborated its association with renal SLE [12,61]. IP-10 is a member of the T-helper 1 lymphocyte chemokines; it is secreted in response to IFN stimulation and has been shown to be involved in the lymphocyte trafficking into afflicted organs in murine lupus models and SLE patients [81].

MCP-1 is another low molecular weight CC chemokine implicated in the recruitment of leukocytes and a mediator of inflammation and injury in LN [30,82]. MCP-1 expression is upregulated by several proinflammatory cytokines, including those belonging to the TNF superfamily. It has been shown that the TNF𝛼/TNF receptor II (TNFRII) and TWEAK/Fn14 axes both induce the production of MCP-1, which, in turn, amplifies the inflammatory response [30,72,83]. This provides biological advocacy for a link between MCP-1 and other widely investigated biomarkers of LN, such as TWEAK and TNFRII [30,56,83]. MCP-1 was evaluated in several studies [11,30,41,65,84,85,86,87,88,89,90], and its value as a urinary marker of LN activity was further strengthened in a meta-analysis conducted by Xia et al., which reported a pooled sensitivity of 89% and pooled specificity of 63% (AUC = 0.90; pooled OR = 19.4) for distinguishing patients with active LN from patients with inactive LN [91]. Moreover, Urrego-Callejas et al. found that the urine levels of MCP-1 were significantly increased in patients with a National Institutes of Health (NIH) renal pathology chronicity index (CI) score ≥ 4, fibrous crescents, tubular atrophy, and interstitial fibrosis, underscoring the potential utility of this molecule as a less invasive, complementary biomarker of kidney damage in real-life clinical settings [92].

Angiogenesis-related proteins are emerging as a novel putative group of interest in LN. Large proteomic studies identified angiopoietin-like protein 4 (Angptl4) and angiostatin as useful urinary biomarkers for tracking renal disease activity and kidney pathology in patients with SLE [11,12,80]. Angptl4 is a cytokine implicated in multiple physiological and pathological vascular processes. Vanarsa et al. found that Angptl4 could differentiate between patients with active LN and active non-renal SLE, while it also strongly correlated with rSLEDAI (AUC = 0.96; r = 0.66; *p* < 0.001) [80]. Angiostatin is a 38-kDa peptide derived from the proteolytic cleavage of plasminogen and/or plasmin, with prominent inhibitory effects on angiogenesis and endothelial cell proliferation [93,94]. Several enzymes, including plasmin, plasminogen activators, and other proteinases, e.g., matrix metalloproteinase (MMP)-2, MMP3, MMP-7, and MMP-9, have been reported to mediate the production of angiostatin and angiostatin-like molecules [93]. Angiostatin demonstrated an excellent association with LN in different studies (AUC = 0.95–0.99; *p* < 0.001 for all) [12,69,95]. Moreover, angiostatin has been reported to correlate with several disease activity and organ damage measures, including rSLEDAI, SLEDAI, the NIH renal histology activity index (AI), and NIH renal pathology CI (r = 0.33–0.52, r = 0.36, r = 0.97, and r = 0.52, respectively; *p* < 0.001 for all), thus emerging as a promising versatile marker with potential value in LN surveillance [69,95].

LN has been associated with a hypercoagulability state as a result of the inflammatory response, and renal thrombotic angiopathy is a feature of severe kidney disease activity in patients with SLE [96]. Qin et al. conducted an exploratory study to evaluate the potential usefulness of haemostasis-related proteins as urinary markers of LN [97]. Among the proteins assessed, i.e., the d-dimer, tissue factor, tissue factor pathway inhibitor (TFPI), and plasmin and urinary plasmin outperformed the others, showing the strongest correlation with rSLEDAI and the Systemic Lupus International Collaborating Clinics Renal Activity Score (SLICC-RAS) (r = 0.50 and r = 0.58, respectively; *p* < 0.001), as well as the ability to distinguish active from inactive LN (AUC = 0.86; *p* < 0.001) [97]. Although it still is poorly understood whether urinary plasmin derives from serum plasmin or originates from kidney tissue, it has been demonstrated that the renal expression of its autocatalytic product, angiostatin, is increased in patients with LN [69,97]. In addition, macrophages are likely to contribute to chronic kidney damage and fibrous crescent formation by means of the expression of procoagulant factors [92,96], thus reinforcing the hypothesis of the renal origin of urinary plasmin.

Cell adhesion molecules (CAMs) are key mediators of the inflammatory process and play a critical role in leukocyte transmigration across the endothelium within affected tissues by interacting with integrins expressed on the surfaces of leukocytes [98]. Among them, urinary vascular cell adhesion molecule 1 (VCAM-1) and activated leukocyte cell adhesion molecule (ALCAM) are broadly validated LN biomarkers [11,66,67,68,87,95,99,100], both displaying a good ability to distinguish patients with active LN from patients with inactive LN or non-renal SLE, and correlate with markers of clinical renal disease activity (see Table 2 for the detailed metrics). Urinary VCAM-1 holds promise as a non-invasive predictor of underlying active renal disease; a strong association between urinary VCAM-1 and NIH renal histology AI was displayed in two separate studies (r = 0.42 and r = 0.97; *p* = 0.05 and *p* < 0.001, respectively) [66,95]. Moreover, the urine levels of VCAM-1 were found to correlate with NIH renal pathology CI and the chronic kidney disease (CKD) stages (r = 0.30 and r = 0.39–0.50; *p* < 0.05 for all), suggesting a role as a marker of organ damage [87,99]. Additionally, urinary ALCAM demonstrated a good ability to differentiate between proliferative and membranous LN (AUC = 0.81; *p* < 0.001), outperforming the ability of traditional markers, i.e., C3, C4, anti-dsDNA, and 24-h urinary protein excretion, in a recent study by Ding et al. [68].

CD163 is a scavenger receptor expressed on the surface of alternatively activated M2c macrophages [101]. Urinary soluble CD163 (sCD163) originates from the extracellular portion of its membrane-bound counterpart upon cleavage by MMPs during inflammation [102]. Soluble CD163 is considered a yardstick of glomerular infiltration of CD163^+^ M2 macrophages, which, in turn, is linked to LN histological and clinical renal activity [102,103]. While having a physiological function in wound healing and tissue repair, this subset of macrophages was also found to be increased in areas of glomerular and tubular injury and may be a major driver of crescent formation and interstitial fibrosis [101,103]. When evaluated in multiple independent cohorts [79,101,102,103], urinary sCD163 could discriminate between patients with active LN from patients with inactive LN or non-renal SLE, suggesting a potential use in monitoring LN disease activity (see Table 2 and Table 3 for the detailed metrics). In addition, in a study by Endo et al., urinary sCD163 was shown to be a reliable predictor of renal proliferative disease in patients with LN (AUC = 0.83–0.89; sens.: 83%; spec.: 86%) [102]. Similar results were obtained in another study where urinary sCD163 outperformed the conventional markers, i.e., anti-dsDNA, C3, C4, and uPCR, in distinguishing proliferative from non-proliferative LN (AUC = 0.89; *p* < 0.001) [103].

Furthermore, along with their diagnostic utility, the levels of TWEAK and NGAL were also found to resemble clinical disease activity in patients with LN, placing these molecules among the candidate biomarkers for LN surveillance (Table 2).

The metrics of the selected biomarkers of clinical disease activity, histological disease activity, and organ damage in LN, as determined from the present systematic literature review, are provided in Table 2, Table 3, and Table 4, respectively.

**Table 2:** Performances of selected biomarkers of clinical disease activity in LN.

Biomarker	Sample	Comparator	Disease activity	Metrics	References
Autoantibodies					
Anti-C1q (+)	Serum/ Plasma	N/A	SLEDAI; ECLAM	r = 0.47 (SLEDAI); r = 0.28 (ECLAM)	Bock et al., 2015 [104]
		Inactive LN	proteinuria; SLEDAI	AUC = 0.76; sens.: 72%; spec.: 55%; r = 0.28 (proteinuria); r = 0.28 (SLEDAI)	Gómez-Puerta et al., 2018 [41]
		Inactive LN	proteinuria; active urinary sediment	AUC = 0.73; sens.: 63%; spec.: 75%; PPV: 69%; NPV: 67%; OR = 5.1	Kianmehr et al., 2021 [105]
		Inactive LN	proteinuria; active urinary sediment	OR = 8.4	Sjöwall et al., 2018 [50]
		SLE with no renal flares	renal flares	sens.: 70%; spec.: 44%	Birmingham et al., 2016 [51]
		SLE with no renal flare	renal flares	sens.: 75%; spec.: 69%; PPV: 35%; NPV: 93%; HR_adj_ = 1.1	Fatemi et al., 2016 [106]
Anti-dsDNA (+)	Serum/ Plasma	Inactive LN	proteinuria; SLEDAI	AUC = 0.88; sens.: 71%; spec.: 88%	Jakiela et al., 2018 [61]
		Inactive LN	proteinuria; active urinary sediment; SLEDAI	AUC = 0.70; sens.: 71%; spec.: 63%; PPV: 63%; NPV: 71%; OR = 4.2 r = 0.23 (SLEDAI)	Kianmehr et al., 2021 [105]
		Inactive LN	proteinuria; active urinary sediment	OR = 4.8	Sjöwall et al., 2018 [50]
		SLE with no renal flares	renal flares	AUC = 0.85; sens.: 88%; spec.: 83%; PPV: 43%; NPV: 97%; HR = 21.7	Fasano et al., 2020 [107]
PTEC-binding IgG (+)	Serum/Plasma	Inactive LN	renal flares	AUC = 0.63; sens.: 46%; spec.: 80%; PPV: 44%; NPV: 81%	Yap et al., 2016 [108]
**Complements**					
C3 (low)	Serum/ Plasma	Inactive LN	proteinuria; SLEDAI	AUC = 0.88; sens.: 100%; spec.: 65%	Jakiela et al., 2018 [61]
		N/A	SLEDAI	r = −0.99 (SLEDAI)	Selvaraja et al., 2019 [109]
		Active non-renal SLE	renal flares	sens.: 70%; spec.: 59%; OR = 2.5	Ruchakorn et al., 2019 [110]
		SLE with no renal flares	renal flares	AUC = 0.76; sens.: 100%; spec.: 51%; PPV: 23%; NPV: 100%; HR = 6.0	Fasano et al., 2020 [107]
C4 (low)	Serum/ Plasma	N/A	SLEDAI	r = −0.83 (SLEDAI)	Selvaraja et al., 2019 [109]
		Inactive LN	proteinuria; SLEDAI	AUC = 0.88; sens.: 81%; spec.: 88%	Jakiela et al., 2018 [61]
		SLE with no renal flares	renal flares	AUC = 0.82; sens.: 100%; spec.: 62%; PPV: 28%; NPV: 100%; HR = 5.5	Fasano et al., 2020 [107]
		SLE with no renal flares	renal flares	OR_adj_ = 5.6	Buyon et al., 2017 [111]
**Kidney disease-related markers**					
Proteinuria (**↑**) (>500 mg/24 h)	Urine	Inactive LN	proteinuria; active urinary sediment	AUC = 0.94	Dolff et al., 2013 [112]
		Inactive LN	proteinuria; SLEDAI	AUC = 0.99; sens.: 88%; spec.: 100%	Jakiela et al., 2018 [61]
		SLE with no renal flares	renal flares	PPV: 43%; NPV: 85%; HR_adj_ = 1.1	Fatemi et al., 2016 [106]
WBC (**↑**)	Urine	Inactive LN	proteinuria; SLEDAI	AUC = 0.75; sens.: 71%; spec.: 73%	Jakiela et al., 2018 [61]
RBC (**↑**)	Urine	Inactive LN	proteinuria; SLEDAI	AUC = 0.92; sens.: 77%; spec.: 100%	Jakiela et al., 2018 [61]
Granular casts (+)	Urine	Inactive LN	proteinuria; SLEDAI	AUC = 0.91; sens.: 82%; spec.: 91%	Jakiela et al., 2018 [61]
**Cytokines/chemokines**					
IL-10 (**↑**)	Serum/ Plasma	Inactive LN	proteinuria; SLEDAI	AUC = 0.87; sens.: 71%; spec.: 85%	Jakiela et al., 2018 [61]
		N/A	SLEDAI	r = 0.98 (SLEDAI)	Selvaraja et al., 2019 [109]
IL-17 (**↑**)	Serum/ Plasma	Inactive LN	SLEDAI	AUC = 0.91; r = 0.63 (SLEDAI)	Dedong et al., 2019 [77]
		Inactive LN	BILAG renal score	AUC = 0.81; r = 0.26 (BILAG renal score)	Nordin et al., 2019 [113]
IL-7 (**↑**)	Urine	Inactive SLE	rSLEDAI	AUC = 0.92; sens.: 84%; spec.: 95%; PPV: 95%; NPV: 84%; r = 0.70 (rSLEDAI)	Stanley et al., 2019 [78]
IL-12 p40 (**↑**)				AUC = 0.93; sens.: 87%; spec.: 100%; PPV: 100%; NPV: 88%; r = 0.67 (rSLEDAI)	
IL-15 (**↑**)				AUC = 0.91; sens.: 93%; spec.: 100%; PPV: 100%; NPV: 92%; r = 0.67 (rSLEDAI)	
MCP-1 (**↑**)	Urine	Inactive LN; Non-renal SLE	rSLEDAI	AUC = 0.70; r = 0.35 (rSLEDAI)	Liu et al., 2020 [87]
		Inactive LN	SLEDAI	AUC = 0.76; sens.: 81%; spec.: 85%	Bona et al., 2020 [86]
		Inactive SLE	rSLEDAI	AUC = 0.79; sens.: 93%; spec.: 68%; PPV: 93%; NPV: 68%	Stanley et al., 2020 [11]
		Inactive LN	proteinuria; rSLEDAI	AUC = 1.00; sens.: 100%; spec.: 100%; PPV: 100%; NPV:100%; r = 0.84 (proteinuria); r = 0.92 (rSLEDAI)	Elsaid et al., 2021 [30]
		Inactive LN	SLEDAI-2K	AUC = 0.81; sens.: 50%; spec.: 90%; r = 0.39 (SLEDAI-2K)	Rosa et al., 2012 [84]
		Inactive LN	proteinuria; SLEDAI	AUC = 0.71; sens.: 70%; spec.: 58%; r = 0.47 (proteinuria); r = 0.33 (SLEDAI)	Gómez-Puerta et al., 2018 [41]
		Inactive LN	N/A	AUC = 0.90; sens.: 89%; spec.: 63%; OR = 19.4	Xia et al., 2020 [91]
IP-10/CXCL10 (**↑**)	Urine	Inactive SLE	rSLEDAI	AUC = 0.94; sens.: 87–88%; spec.: 81–100%; PPV: 100%; NPV: 88%; r = 0.67–0.74 (rSLEDAI)	Stanley et al., 2019 [78]
		Inactive LN	proteinuria; SLEDAI	AUC = 0.93; sens.: 88%; spec.: 81%	Jakiela et al., 2018 [61]
		Healthy controls	N/A	AUC = 0.92	Zhang et al., 2020 [12]
PF-4 (**↑**)	Urine	Inactive SLE	rSLEDAI	AUC = 0.71–0.88; sens.: 54–93%; spec.: 79–96%; PPV: 82–94%; NPV: 77–88%	Stanley et al., 2020 [11]
TARC (**↑**)	Urine	Inactive SLE	rSLEDAI	AUC = 0.91; sens.: 78%; spec.: 92%, PPV: 91%; NPV: 80%; r = 0.70 (rSLEDAI)	Stanley et al., 2019 [78]
TGFβ1 (**↑**)	Urine	Active non-renal SLE	proteinuria	r = 0.51 (proteinuria)	Fava et al., 2022 [79]
		Active non-renal SLE	rSLEDAI	AUC = 0.78; r = 0.37 (rSLEDAI)	Vanarsa et al., 2020 [80]
TWEAK (**↑**)	Urine	N/A	proteinuria	r = 0.61 (proteinuria)	Reyes-Martínez et al., 2018 [27]
		Inactive LN	proteinuria; rSLEDAI	AUC = 1.00; sens.: 100%; spec.: 100%; PPV: 100%; NPV: 100%; r = 0.84 (proteinuria); r = 0.89 (rSLEDAI)	Elsaid et al., 2021 [30]
**Angiogenesis-related molecules**					
Angptl4 (**↑**)	Urine	Active non-renal SLE	rSLEDAI	AUC = 0.96; r = 0.66	Vanarsa et al., 2020 [80]
		Healthy controls	N/A	AUC = 0.92	Zhang et al., 2020 [12]
Angiostatin (**↑**)	Serum/ Plasma	Inactive LN	SLEDAI	AUC = 0.83	Wu et al., 2016 [13]
	Urine	Inactive LN	rSLEDAI	AUC = 0.99; sens.: 83%; spec.: 100%; r = 0.33 (rSLEDAI)	Soliman et al., 2017 [95]
		Healthy controls	N/A	AUC = 0.97	Zhang et al., 2020 [12]
		Inactive SLE	rSLEDAI; SLEDAI; SLICC-RAS	AUC = 0.83 r = 0.52 (rSLEDAI); r = 0.36 (SLEDAI); r = 0.68 (SLICC-RAS)	Wu et al., 2013 [69]
**Haemostasis-related molecules**					
Plasmin (**↑**)	Urine	Inactive LN	rSLEDAI; SLICC-RAS	AUC = 0.86; sens.: 100%; spec.: 70%; PPV: 96%; NPV: 50%; r = 0.50 (rSLEDAI); r = 0.58 (SLICC-RAS)	Qin et al., 2019 [97]
Tissue Factor (**↑**)				AUC = 0.74; sens.: 61%; spec.: 85%; PPV: 90%; NPV: 35%; r = 0.33 (rSLEDAI); r = 0.38 (SLICC-RAS)	
TFPI (**↑**)				AUC = 0.77; sens.: 86%; spec.: 58%; PPV: 92%; NPV: 36%; r = 0.40 (rSLEDAI); r = 0.31 (SLICC-RAS)	
		Inactive SLE	rSLEDAI	AUC = 0.71–0.88; sens.: 57–80%; spec.: 84–89%; PPV: 73–89%; NPV: 73–82%	Stanley et al., 2020 [11]
**Cell adhesion molecules**					
ALCAM (**↑**)	Urine	N/A	rSLEDAI	r = 0.35–0.41 (rSLEDAI)	Chalmers et al., 2022 [67]
		Inactive SLE	rSLEDAI	AUC = 0.84% sens.: 79–94%; spec.: 70–95%; PPV: 86–91%; NPV: 90–92%	Stanley et al., 2020 [11]
		N/A	rSLEDAI; SLICC-RAS	r = 0.55 (rSLEDAI); r = 0.58 (SLICC-RAS)	Ding et al., 2020 [68]
ICAM-1 (**↑**)	Urine	Inactive LN	proteinuria; active urinary sediment	AUC = 0.97; sens.: 93–98%; spec.: 81–86%	Wang et al., 2018 [114]
		SLE with no renal flares	renal flares	AUC = 0.75; sens.: 88%; spec.: 59%; PPV: 25%; NPV: 97%; HR = 8.5	Fasano et al., 2020 [107]
NCAM-1 (**↑**)	Urine	Inactive LN	proteinuria; active urinary sediment	AUC = 0.88; sens.: 82%; spec.: 87%	Wang et al., 2018 [114]
		Healthy controls	N/A	AUC = 0.75	Zhang et al., 2020 [12]
VCAM-1 (**↑**)	Serum/ Plasma	Inactive LN	rSLEDAI-2K; SLEDAI-2K	AUC = 0.86; sens.: 69%; spec.: 90%; r = 0.61 (rSLEDAI-2K); r = 0.62 (SLEDAI-2K)	Yu et al., 2021 [115]
	Urine	N/A	rSLAM-R	r = 0.26 (rSLAM-R)	Howe et al., 2012 [100]
		Inactive SLE	rSLEDAI	AUC = 0.84–0.87; sens.:92–96%; spec.: 65–74%; PPV: 93–95%; NPV: 60–72%	Stanley et al., 2020 [11]
		N/A	rSLEDAI	r = 0.55 (rSLEDAI)	Liu et al., 2020 [87]
		SLE with no renal flares	renal flares	AUC = 0.76; sens.: 75%; spec.: 75%; PPV: 32%; NPV: 95%; HR = 7.5	Fasano et al., 2020 [107]
		Inactive LN	rSLEDAI; SLEDAI	AUC = 0.98; sens.: 100%; spec.: 90%; r = 0.32 (rSLEDAI); r = 0.32 (SLEDAI)	Soliman et al., 2017 [95]
**Other proteins**					
Axl (**↑**)	Serum/ Plasma	Inactive LN	SLEDAI	AUC = 0.87	Wu et al., 2016 [13]
Calpastatin (**↑**)	Urine	Inactive SLE	rSLEDAI	AUC = 0.72–0.75; sens.: 50–66%; spec.: 78–100%; PPV: 75–82%; NPV: 70–100%	Stanley et al., 2020 [11]
CD163 (**↑**)	Urine	Inactive LN	N/A	AUC = 0.98–0.99; sens.: 97%; spec.: 94%	Mejia-Vilet et al., 2020 [101]
		Active non-renal SLE	rSLEDAI	AUC = 0.87–0.94; r = 0.45–0.75 (rSLEDAI)	Zhang et al., 2020 [103]
		N/A	proteinuria	r = 0.40 (proteinuria)	Fava et al., 2022 [79]
Ferritin (**↑**)	Serum/ Plasma	Inactive LN	SLEDAI	AUC = 0.84	Wu et al., 2016 [13]
FOLR2 (**↑**)	Urine	Active non-renal SLE	rSLEDAI	AUC = 0.73; r = 0.62 (rSLEDAI)	Vanarsa et al., 2020 [80]
Hemopexin (**↑**)	Urine	Inactive SLE	rSLEDAI	AUC = 0.73–0.80; sens.: 85–100%; spec.: 56–99%; PPV: 79–100%; NPV: 57–70%	Stanley et al., 2020 [11]
IGFBP-2 (**↑**)	Serum/ Plasma	N/A	rSLEDAI	r = 0.41 (rSLEDAI)	Ding et al., 2016 [71]
L-selectin (**↑**)	Urine	Active non-renal SLE	rSLEDAI	AUC = 0.86; r = 0.73 (rSLEDAI)	Vanarsa et al., 2020 [80]
NGAL (**↑**)	Urine	Inactive LN	rSLEDAI-2K	AUC = 0.83; sens.: 89%; spec.: 67%	Alharazy et al., 2013 [116]
		Inactive LN	proteinuria; SLEDAI	AUC = 0.67; sens.: 70%; spec.: 62%; r = 0.40 (proteinuria); r = 0.30 (SLEDAI)	Gómez-Puerta et al., 2018 [41]
		N/A	rSLEDAI	r = 0.42 (rSLEDAI)	Liu et al., 2020 [87]
PDGFRβ (**↑**)	Urine	Active non-renal SLE	rSLEDAI	AUC = 0.67	Vanarsa et al., 2020 [80]
Peroxiredoxin 6 (**↑**)	Urine	Inactive SLE	rSLEDAI	AUC = 0.64–0.75; sens.: 50–56%; spec.: 79–91%; PPV: 68–87%; NPV: 64–68%	Stanley et al., 2020 [11]
Progranulin (**↑**)	Serum/ Plasma	Stable LN	rSLEDAI; SLEDAI	AUC = 0.88; sens.: 53%; spec.: 89%; PPV: 82%; NPV: 66%	Wu et al., 2016 [117]
		Non-LN renal disorder		AUC = 0.67; sens.: 60%; spec.: 100%; PPV: 100%; NPV: 73%; r = 0.57 (rSLEDAI); r = 0.62 (SLEDAI)	
	Urine	Inactive LN		AUC = 0.90; sens.: 65%; spec.: 99%; PPV: 98%; NPV: 74%; r = 0.59 (rSLEDAI); r = 0.58 (SLEDAI)	
Properdin (**↑**)	Urine	Inactive SLE	rSLEDAI	AUC = 0.71–0.85; sens.: 62–86%; spec.: 84–90%; PPV: 79–90%; NPV: 68–86%	Stanley et al., 2020 [11]
RBP4 (**↑**)	Urine	SLE with no proteinuric flare	proteinuric flares	AUC = 0.67; sens.: 93%; spec.: 67%; HR = 9.5	Go et al., 2018 [118]
		N/A	rSLEDAI; SLEDAI; uPCR	r = 0.31 (rSLEDAI); r = 0.31 (SLEDAI); r = 0.39 (uPCR)	Aggarwal et al., 2017 [119]
SDC-1 (**↑**)	Serum/ Plasma	Inactive LN	proteinuria; rSLEDAI-2K; SLEDAI-2K	AUC = 0.91; sens.: 85%; spec.: 86%; r = 0.57 (proteinuria); r = 0.68 (rSLEDAI-2K); r = 0.54 (SLEDAI-2K)	Yu et al., 2021 [120]
		N/A	SLEDAI; uPCR	r = 0.60 (SLEDAI); r = 0.45 (uPCR)	Kim et al., 2015 [121]
sTNFRII (**↑**)	Serum/ Plasma	Non-renal SLE	rSLEDAI; rLAI	AUC = 0.77; r = 0.30 (rSLEDAI); r = 0.39 (rLAI)	Smith et al., 2019 [122]
		Inactive LN	N/A	AUC = 0.81	Wu et al., 2016 [13]
TSP1 (**↑**)	Urine	Active non-renal SLE	rSLEDAI	AUC = 0.72	Vanarsa et al., 2020 [80]
TTP1 (**↑**)	Urine	Active non-renal SLE	rSLEDAI	AUC = 0.84	Vanarsa et al., 2020 [80]
**Microparticles**					
MP-HMGB1^+^ (**↑**)	Urine	Inactive LN	N/A	AUC = 0.83; sens.: 55%; spec.: 93%	Burbano et al., 2019 [48]

Biomarkers are structured into subgroups (highlighted in bold) based on clinical/functional affinities. ALCAM: activated leukocyte cell adhesion molecule; Angptl4: angiopoietin-like protein 4; Anti-dsDNA: anti-double-stranded DNA; AUC: area under the curve; BILAG: British Isles Lupus Assessment Group; CCL: C-C motif chemokine ligand; Cr: creatinine; CXCL: C-X-C motif chemokine ligand; C3: complement component 3; C4: complement component 4; ECLAM: European Consensus Lupus Activity Measurement; FOLR2: folate receptor beta; HMGB1: high mobility group box 1; HR: hazard ratio; HR_adj_: adjusted hazard ratio; ICAM-1: intercellular cell adhesion molecule 1; IGFBP-2: insulin-like growth factor binding protein 2; IP-10/CXCL10: interferon gamma inducible protein-10/C-X-C motif chemokine ligand 10; LN: lupus nephritis; MCP-1: monocyte chemoattractant protein 1; MP: microparticle; N/A: not applicable; NCAM-1: neural cell adhesion molecule 1; NGAL: neutrophil gelatinase associated lipocalin; NPV: negative predictive value; OR: odds ratio; PDGFRβ: platelet-derived growth factor receptor beta; PF-4: platelet factor 4; PPV: positive predictive value; PTEC-binding IgG: proximal renal tubular epithelial cell-binding immunoglobulin G; r: correlation coefficient; RBC: red blood cells; RBP4: retinol binding protein 4; rLAI: renal Lupus Activity Index; rSLEDAI: renal SLEDAI; rSLEDAI-2K: renal SLEDAI 2000; SDC-1: syndecan 1; sens.: sensitivity; sIL-7R: soluble interleukin 7 receptor; SLAM-R: Systemic Lupus Activity Measure Revised; SLE: systemic lupus erythematosus; SLEDAI: Systemic Lupus Erythematosus Disease Activity Index; SLICC: Systemic Lupus International Collaborating Clinics; SLICC-RAS: Systemic Lupus International Collaborating Clinics Renal Activity Score; spec.: specificity; sTNFRII: soluble tumour necrosis factor alpha receptor II; TARC: thymus- and activation-regulated chemokine; TFPI: tissue factor pathway inhibitor; TGFβ1: transforming growth factor β_1_; TSP1: thrombospondin 1; TTP1: tripeptidyl-peptidase 1; TWEAK: TNF-like weak inducer of apoptosis; uPCR: urine protein to creatinine ratio; VCAM-1: vascular cell adhesion molecule 1; WBC: white blood cells; (+): positivity; **↑**: elevated.

**Table 3:** Performances of selected biomarkers of histological disease activity.

Biomarker	Sample	Comparator	Disease activity	Metrics	References
Complement					
C1q (low)	Serum/ Plasma	N/A	AI	r = −0.33 (AI)	Tan et al., 2013 [123]
C3 (low)	Serum/ Plasma	membranous LN	proliferative LN	AUC = 0.77; sens.: 75%; spec.: 74%; PPV: 92%; NPV: 44%	Ding et al., 2020 [68]
**Kidney disease-related markers**					
Proteinuria (**↑**) (>500 mg/24 h)	Urine	Inactive LN	proliferative LN	AUC = 0.91; sens.: 89%; spec.: 85%	Enghard et al., 2014 [124]
**Cytokines/chemokines**					
IL-17 (**↑**)	Serum/ Plasma	N/A	AI	r = 0.52 (AI)	Dedong et al., 2019 [77]
IL-16 (**↑**)	Urine	N/A	AI	r = 0.59–0.73 (AI)	Fava et al., 2022 [79]
MCP-1 (**↑**)	Urine	Non-proliferative LN	proliferative LN	AUC = 0.64–0.78	Endo et al., 2016 [102]
TGFβ1 (**↑**)	Urine	N/A	AI	r = 0.65 (AI)	Fava et al., 2022 [79]
**Angiogenesis-related molecules**					
Angiostatin (**↑**)	Urine	N/A	AI	r = 0.93 (AI)	Soliman et al., 2017 [95]
**Cell adhesion molecules**					
ALCAM (**↑**)	Urine	Membranous LN	proliferative LN	AUC = 0.81; sens.: 78%; spec.: 81%; PPV: 94%; NPV: 52%	Ding et al., 2020 [68]
VCAM-1 (**↑**)	Urine	N/A	AI	r = 0.42 (AI)	Singh et al., 2012 [66]
		N/A	AI	r = 0.97 (AI)	Soliman et al., 2017 [95]
**Other proteins**					
CD163 (**↑**)	Urine	N/A	AI	r = 0.48–0.59 (AI)	Mejia-Vilet et al., 2020 [101]
		Non-proliferative LN	proliferative LN	AUC = 0.83–0.89; sens.: 83%; spec.: 86%	Endo et al., 2016 [102]
		N/A	AI	r = 0.41 (AI)	
		Non-proliferative LN	proliferative LN	AUC = 0.89	Zhang et al., 2020 [103]
		N/A	AI	r = 0.40 (AI)	
		N/A	AI	r = 0.67 (AI)	Fava et al., 2022 [79]
SDC-1 (**↑**)	Serum/ Plasma	N/A	AI	r = 0.63; r_adj_ = 0.66 (AI)	Kim et al., 2015 [121]
sTNFRII (**↑**)	Serum/ Plasma	N/A	AI	r = 0.40 (AI)	Wu et al., 2016 [13]
**Renal tissue markers**					
CSF-1 (**↑**)	Kidney biopsy	Non-renal SLE	AI	r = 0.46	Menke et al., 2015 [125]

Biomarkers are structured into subgroups (highlighted in bold) based on clinical/functional affinities. AI: National Institutes of Health (NIH) renal histology activity index; ALCAM: activated leukocyte cell adhesion molecule; AUC: area under the curve; CSF-1: colony stimulating factor 1; C3: complement component 3; C1q: complement component 1q; LN: lupus nephritis; MCP-1: monocyte chemoattractant protein 1; N/A: not applicable; NPV: negative predictive value; PPV: positive predictive value; r: correlation coefficient; r_adj_: adjusted correlation coefficient; SDC-1: syndecan 1; sens.: sensitivity; spec.: specificity; sTNFRII: soluble tumour necrosis factor alpha receptor II; TGFβ1: transforming growth factor β_1_; VCAM-1: vascular cell adhesion molecule 1; **↑**: elevated.

**Table 4:** Performances of selected biomarkers of organ damage in LN.

Biomarker	Sample	Comparator	Organ Damage	Metrics	References
Autoantibodies					
Anti-dsDNA (+)	Serum/Plasma	Non-CKD SLE	CKD stages	OR_adj_ = 2.0	Barnado et al., 2019 [57]
**Kidney disease-related markers**					
Urea (**↑**) (>10.25 mmol/L)	Serum/Plasma	Non-CKD LN	CKD stages	AUC = 0.91; sens.: 85%; spec.: 83%; PPV: 82%; NPV: 86%	Yang et al., 2016 [23]
**Other proteins**					
Angiostatin (**↑**)	Urine	N/A	CI	r = 0.52	Wu et al., 2013 [69]
IGFBP-2 (**↑**)	Serum/Plasma	N/A	CI	r = 0.58	Ding et al., 2016 [71]
IGFBP-4 (**↑**)	Serum/Plasma	N/A	CI; eGFR	r = 0.71; r = −0.62	Wu et al., 2016 [126]
Resistin (**↑**)	Serum/Plasma	N/A	creatinine; BUN	r = 0.45; r = 0.54	Hutcheson et al., 2015 [127]
sTNFRII (**↑**)	Serum/Plasma	N/A	CI	r = 0.34–0.43	Parodis et al., 2017 [83]
		N/A	CI; eGFR	r = 0.57; r = −0.50	Wu et al., 2016 [13]
VCAM-1 (**↑**)	Urine	N/A	CKD stages	r = 0.39–0.50	Parodis et al., 2020 [99]
		N/A	CI	r = 0.30	Liu et al., 2020 [87]
**Renal tissue markers**					
Periostin (**↑**)	Kidney biopsy	N/A	CI; creatinine; BUN; eGFR	r = 0.59; r = 0.43; r = 0.31; r = −0.45	Wantanasiri et al., 2015 [128]

Biomarkers are structured into subgroups (highlighted in bold) based on clinical/functional affinities. Anti-dsDNA: anti-double-stranded DNA; AUC: area under the curve; BUN: blood urea nitrogen; CKD: chronic kidney disease; CI: NIH renal pathology chronicity index; eGFR: estimated glomerular filtration rate; IGFBP-2: insulin-like growth factor binding protein 2; IGFBP-4: insulin-like growth factor binding protein 4; LN: lupus nephritis; N/A: not applicable; NPV: negative predictive value; OR: odds ratio; OR_adj_: adjusted odds ratio; PPV: positive predictive value; r: correlation coefficient; sens.: sensitivity; SLE: systemic lupus erythematosus; spec.: specificity; sTNFRII: soluble tumour necrosis factor receptor II; VCAM-1: vascular cell adhesion molecule 1; (+): positivity; **↑**: elevated.

### 3.3. Biomarkers of Response to Therapy

Axl is a tyrosine kinase receptor, which has been suggested to be involved in LN by means of mediating mesangial proliferation through interaction with its ligand, Gas6 [129]. In addition, when bound to Gas6, Axl plays an important immunoregulatory role on innate immune cells and promotes the clearance of apoptotic bodies, a process that is well-known to be impaired in SLE [130]. A soluble form of Axl (sAxl) is obtained from the ectodomain of Axl, which is present on the surfaces of macrophages and B cells, through proteolytic cleavage [56]. The shedding of sAxl, enhanced by inflammation, may be instrumental in LN pathogenesis by dysregulating physiological Axl/Gas6 signalling. Specifically, sAxl may act as a decoy receptor and block Gas6-induced anti-inflammatory effects on immune cells [131]. There is evidence of a correlation between serum sAxl and SLE activity [132], and in a recent discovery study, sAxl could differentiate between active LN and non-renal SLE [13]. In a prospective cohort of biopsy-proven LN, lower post-treatment serum levels of sAxl were observed in clinical responders but not in clinical non-responders and predicted good long-term renal outcomes [131]. Additionally, in the same work, high baseline levels of sAxl were strongly associated with the histological response to therapy based on post-treatment repeat biopsies after adjustment for the confounding factors (OR_adj_ = 9.3; *p* = 0.02) [131].

A B-cell-activating factor belonging to the TNF ligand superfamily (BAFF) is a cytokine member of the TNF family that is believed to have a key role in SLE pathogenesis and has also been suggested as a promising biomarker for LN [133]. The importance of BAFF in LN was recently corroborated with approval by the regulatory authorities of the BAFF inhibitor belimumab for the treatment of active LN in adults [134]. In a Swedish LN cohort, low baseline levels of serum BAFF were shown to be predictive of the clinical and histopathological responses to therapy, the latter based on per-protocol repeat biopsies, demonstrating a PPV of 92% for the clinical response in a subgroup of SLE patients with proliferative nephritis [135].

Among the aforementioned biomarkers, urinary MCP-1, NGAL, and CD163 deserve mention for their potential usefulness as indicators of response to therapy, as they have exhibited good predictive performances in multiple studies [35,72,79,87,101,136] (see Table 5 for the detailed metrics).

The identification of kidney tissue-based predictors of response could potentially improve the treatment selection processes and histological assessment of LN. In a retrospective analysis of Japanese patients, Ichinose et al. found that the podocyte foot process width (FPW) could be used to predict the complete renal response 6 and 12 months after the commencement of induction therapy, suggesting the use of FPW as an indicator of abnormality in LN [137].

Another study, albeit comprising a small cohort, demonstrated that glomerular C9 staining was an independent predictor of poor response to treatment 6 and 12 months after the initiation of therapy [138], lending support to the notion that a membrane attack complex (MAC)-mediated injury may be a major driver of tissue injury in LN [139].

Metrics of biomarkers of responses to therapy in LN, as derived from the present review, are summarised in Table 5.

**Table 5:** Performances of selected biomarkers of responses to therapy in LN.

Biomarker	Sample	Main Findings	References
Autoantibodies			
Anti-dsDNA (-) (disappearance at month 6)	Serum/Plasma	Sens.: 70%; spec.: 56%; PPV: 67%; NPV: 59% to predict a CRR by month 12	Mejia-Vilet et al., 2020 [101]
**Complement**			
C3 (**↑**) (normalization or 25% increase at month 6)	Serum/Plasma	Sens.: 65–70%; spec.: 67–72%; PPV: 73–75%; NPV: 62–63% to predict CRR by month 12	Mejia-Vilet et al., 2020 [101]
**Kidney disease-related markers**			
Proteinuria (**↓**) (baseline levels 0.1–0.87 g/24 h)	Urine	Low levels are predictive of CRR at 6 months (OR = 4.3) after immunosuppressive therapy	Ichinose et al., 2018 [137]
uPCR (**↓**) (<1.5 g/g at month 6)	Urine	Sens.: 86%; spec.: 81%; PPV: 81%; NPV: 86% to predict CRR by month 12	Mejia-Vilet et al., 2020 [101]
**Cytokines/chemokines**			
APRIL (**↑**) (baseline levels >4 ng/mL)	Serum/Plasma	Predictive of treatment failure after six months: AUC = 0.71; sens.: 65%; spec.: 87%; PPV: 93%; NPV: 54%	Treamtrakanpon et al., 2012 [140]
BAFF (**↓**) (baseline levels <1.5 ng/mL)	Serum/Plasma	Predictive of clinical (PPV: 87%) and histopathological response (PPV: 83%) (mean follow up: 8.1 months)	Parodis et al., 2015 [135]
IL-8 (**↓**) (baseline levels)	Serum/Plasma	Lower values predictive of treatment response after 1-year: AUC = 0.64	Wolf et al., 2016 [141]
IL-23 (**↓**) (baseline levels)	Serum/Plasma	Predictor for outcome of therapy of induction of remission of active LN: AUC = 0.87	Dedong et al., 2019 [77]
MCP-1 (**↑**) (baseline levels)	Urine	Predictive of response to treatment with rituximab at 6 (OR_adj_ = 2.6) and 12 months (OR_adj_ = 0.6)	Davies et al., 2021 [72]
**Other proteins**			
Axl (**↑**) (baseline levels ≥36.6 ng/mL)	Serum/Plasma	Predictive of histological response: OR = 5.5; OR_adj_ = 9.3. Decreased levels in responders compared with non-responders after induction therapy.	Parodis et al., 2019 [131]
CD163 (**↓**) (<370 ng/mmol at month 6)	Urine	Sens.: 90%; spec.: 87%; PPV: 87%; NPV: 90% to predict a CRR by month 12.	Mejia-Vilet et al., 2020 [101]
CSF-1 (**↓**) (decrease ≥25% after initiation of therapy)	Serum/Plasma	Predictive of response to therapy and remission: PPV: 88%; NPV: 58%	Menke et al., 2015 [125]
HNP1-3 (**↓**) (baseline levels)	Serum/Plasma	Predictive of proteinuria remission (mean follow up of 5.5 years): multivariate hazard = 0.2	Cheng et al., 2015 [142]
IL-2Rα (**↓**) (baseline levels)	Serum/Plasma	Low levels are predictive of treatment response after 1-year: AUC = 0.63	Wolf et al., 2016 [141]
NGAL (**↓**) (baseline levels <1964.58 ng/mL) (baseline levels <28.08 ng/mL)	Urine	Predictive of renal response after 6-month induction therapy: AUC = 0.78; sens.: 81%; spec.: 83%; PPV: 56%; NPV: 95%	Liu et al., 2020 [87]
		Discrimination between complete/partial response and non-response after 6-month of induction therapy: AUC = 0.77; sens.: 73%; spec.: 68%	Satirapoj et al., 2017 [35]
NRP-1 (**↑**) (baseline levels >1143 ng/mg Cr)	Urine	High baseline levels are predictive of clinical response; AUC = 0.84; sens.: 87%; spec.: 72%; PPV: 88%; NPV: 71%	Torres-Salido et al., 2019 [143]
OPG (**↓**) (baseline levels)	Serum/Plasma	Low levels are predictive of treatment response after 1-year: AUC = 0.67	Wolf et al., 2016 [141]
RBP4 (**↓**) (baseline leveles <800 ng/mgCr)	Urine	Low levels are predictive of proteinuria remission within 12 months of immunosuppressive therapy in active LN patients: AUC = 0.81; sens.: 82%; spec.: 89%	Go et al., 2018 [118]
sTNFRII (**↑**) (baseline levels >8.6 ng/mL) (baseline levels >9.0 ng/mL)	Serum/Plasma	Predictive of clinical (AUC = 0.86; sens.: 86%; spec.: 80%) and histological response (AUC = 0.90; sens.: 83%; spec.: 80%) among patients with membranous LN (mean follow up: 7.7 months)	Parodis et al., 2017 [83]
S100A8/A9 (**↑**) (baseline levels)	Serum/Plasma	Differences in disease activity (no response vs. “showing improvement”) in response after 6 months of rituximab: OR_adj_ = 0.3 for both	Davies et al., 2020 [144]
S100A12 (**↑**) (baseline levels)			
TF (**↑**) (baseline levels)	Urine	Predictive of response to treatment with rituximab at 12 months (OR_adj_ = 1.4)	Davies et al., 2021 [72]
**Lymphocytes/immunoglobulins**			
IgM (**↑**) (baseline levels 87.5–402 mg/dL)	Serum/Plasma	High levels are predictive of CRR at 12 months (OR = 2.1) after immunosuppressive therapy	Ichinose et al., 2018 [137]
Lymphocyte count (**↑**) (baseline levels 1327–2683/μL)	Serum/Plasma	High levels are predictive of CRR at 12 months (OR = 2.4) after immunosuppressive therapy	Ichinose et al., 2018 [137]
**MicroRNAs**			
miRNA-31-5p (**↑**) (upregulated at flare time and at month 12)	Urine	Significantly upregulated in responder group compared to non-responders: flare time: AUC = 0.68; 12 months after treatment: AUC = 0.76	Garcia-Vives et al., 2020 [145]
miRNA-107 (**↑**) (upregulated at flare time and at month 12)		Significantly upregulated in responder group compared to non-responders: flare time: AUC = 0.73; 12 months after treatment: AUC = 0.73	
miRNA-135b-5p (**↑**) (upregulated at flare time and at month 12)		Significantly upregulated in responder group compared to non-responders: flare time: AUC = 0.78; sens.: 78%; spec.: 71%; 12 months after treatment: AUC = 0.86; sens.: 81%; spec.: 79%	
**Renal tissue markers**			
C9 (+) (positive staining at baseline)	Kidney biopsy	Positive staining is predictive of poor response at 6 months: OR = 5.4; OR_adj_ = 4.6	Wang et al., 2018 [138]
Podocyte foot process width (**↓**) (baseline levels 498–897 nm)	Kidney biopsy	Smaller width is predictive of CRR after induction therapy at 6 months (OR = 4.9) and 12 months (OR = 5.8) after immunosuppressive therapy	Ichinose et al., 2018 [137]

Biomarkers are structured into subgroups (highlighted in bold) based on clinical/functional affinities. Anti-dsDNA: anti-double-stranded DNA; APRIL: a proliferation-inducing ligand; AUC: area under the curve; BAFF: B-cell-activating factor belonging to the TNF ligand superfamily; CRR: complete renal response; CSF-1: colony stimulating factor 1; C3: complement component 3; C9: complement component 9; HNP1-3: human neutrophil peptide 1-3; HR: hazard ratio; IgM: immunoglobulin M; IL-2Rα: interleukin 2 receptor alpha; LN: lupus nephritis; miRNA: microRNA; MCP-1: monocyte chemoattractant protein 1; NGAL: neutrophil gelatinase associated lipocalin; NPV: negative predictive value; NRP-1: neuropilin 1; OPG: osteoprotegerin; OR: odds ratio; PPV: positive predictive value; RBP4: retinol-binding protein 4; sens.: sensitivity; spec.: specificity; sTNFRII: soluble tumour necrosis factor receptor II; TF: transferrin; uPCR: urine protein to creatinine ratio; **↑**: increased; **↓**: decreased; (-): negativity; (+): positivity.

### 3.4. Prognostic Biomarkers

Proteinuria is the current gold standard among clinical markers of LN surveillance [1], and the proteinuria levels post-treatment have been shown to be a robust predictor of long-term renal outcomes in LN in a series of recent studies [146,147,148,149]. Proteinuria has shown a satisfactory negative predictive value, yet a poorer positive predictive value in predicting renal flares (NPV: 85%) [106], and remission with regard to proteinuria (defined as a value of <0.3 g/g creatinine or dipstick test results of trace or lower in three consecutive urinary protein tests over a period of six months) was found to be an indicator of a good prognosis in patients with diffuse proliferative LN in a Korean cohort (risk ratio (RR) of a composite outcome of mortality and development of end-stage kidney disease (ESKD): 0.2; *p* < 0.05) [150]. This is in conformity with the aforementioned report series, where the proteinuria levels <0.7 to 0.8 g/day were found to be appropriate predictors of a favourable prognosis over a longer term in patients with LN [146,147,148].

Among the traditional immunological biomarkers, low levels of C3 have been shown to be a risk factor for renal failure within 20 years (RR_adj_ = 2.0; *p* = 0.01) in a large cohort of SLE patients [4].

In a previous cohort study, high baseline levels of urinary VCAM-1 and ALCAM were predictive of renal function deterioration defined as a decline in eGFR by ≥25% at the 10-year follow-up, yielding a sensitivity of 91% and 73% and a specificity of 76% and 72%, respectively [99].

Anti-neutrophil cytoplasmic antibodies (ANCAs) comprise a family of antibodies directed against cytoplasmic antigens of neutrophils and are a key feature of specific forms of small-vessel vasculitides. Some of the ANCAs have been shown to be strongly associated with so-called “pauci-immune” glomerulonephritides due to the absence of deposits of immune complexes within the glomerular tuft [151,152]. In two retrospective studies of Chinese patients with LN, Wang et al. explored the clinical relevance of ANCAs in LN [153,154]. In these works, anti-myeloperoxidase (MPO) ANCA was demonstrated to be the most prevalent ANCA in ANCA-positive LN patients. An increased risk of mortality in ANCA-positive LN patients compared with ANCA-negative LN patients was observed both in the first (HR_adj_ = 3.4; *p* = 0.03 [153]) and the second study (RR_adj_ = 3.6; *p* = 0.016 [154]).

Novel kidney tissue-based biomarkers may prove to be promising prognostic tools, complementary to the traditional features assessed for the histopathological classification and characterisation of LN. Despite being rather neglected in the current classification schemes, tubulointerstitial lesions are well-known negative prognostic indicators [155,156,157,158] and have now been suggested for incorporation into the quantitative scoring system of the revised International Society of Nephrology/Renal Pathology Society (ISN/RPS) 2016 classification [159,160]. Vascular lesions have also been shown to have a possible role as predictors of unfavourable renal outcomes in LN [96,161,162,163], but a standardised approach and terminology in the evaluation of vasculopathy in SLE and LN are currently lacking [159]. In a retrospective analysis of 202 biopsy-proven LN patients, Leatherwood et al. demonstrated that interstitial fibrosis and tubular atrophy were strong predictors of ESKD (HR_adj_= 5.2; 95% confidence interval (CI): 2.5–10.6) and death (HR_adj_ = 4.2; 95% CI: 1.3–13.9) at a follow-up of up to 25 years [164]. In the same study, vascular injury was also found to be associated with ESKD (HR_adj_ = 2.1; 95% C.I.: 1.2–3.8), although a statistical significance was not reached after adjustment for the serum creatinine and ISN/RPS class [164].

Recently, two studies investigated how different pathways of complement activation may play a role in predicting long-term outcomes in LN [165,166]. Ding et al. showed that arteriolar C4d deposition and C4d and C3c co-deposition were independent risk factors for a poor renal prognosis, defined as ESKD or doubling of the serum creatinine (HR_adj_ = 2.3 and HR_adj_ = 3.7, respectively; *p* < 0.05 for both) [165]. Moreover, the authors of these works observed an association between arteriolar C4d deposition and renal microvascular lesions, which strengthens the notion of a role as a complement in LN pathogenesis [167,168]. Additionally, Kim et al. found that glomerular C3 deposition without C1q and C4 was predictive of kidney disease progression, arguing for key roles in the alternative complement pathway in LN pathogenesis [166].

A summary of the biomarkers of long-term outcomes in LN is detailed in Table 6.

## 4. Conclusions and Perspectives

The relevance of renal involvement in the global disease burden of SLE is reflected by the extensive research on drug development and novel biomarkers toward the improvement of clinical practice and optimisation of disease outcomes. Several new molecules have been investigated for their prospect as potential diagnostic, monitory, or prognostic biomarkers in LN over the last decade, with encouraging results for several of them. Among these, urinary MCP-1 and NGAL showed an adequate diagnostic ability, as well as the ability to reflect disease activity and predict response to therapy. Moreover, our search suggests that urinary VCAM-1, CD163, and ALCAM may hold promise as versatile biomarkers for LN, with potential implications for diagnostic, monitoring, and prognostic purposes, as their biomarker potential has been repeatedly validated across multiple independent cohorts and laboratories.

Given the complexity and the heterogeneity of LN, it is unlikely that one single biomarker is sufficient to capture its entire spectrum of features. Several studies highlighted the necessity of combining different molecules to improve disease evaluations [18,72,87,107]. The direction we foreshadow is the development of panels that integrate different biomarkers for different purposes, thus achieving the best possible accuracy and precision.

While a kidney biopsy remains the gold standard for the diagnosis and classification of LN [6], only a minority of studies investigated kidney tissue-based biomarkers. Research seems to lean toward fluid-based biomarkers, aiming to implement LN management through less invasive modes. In alignment with this aim, urine constitutes an attractive source for sampling, since it is easily obtainable, non-invasive, and theoretically more specific of kidney involvement than peripheral blood. However, it is important to underline the importance of the integration of tissue-based information in fluid-based biomarker research as a strategy to accurately determine the best peripheral molecular readouts for kidney-specific injury [171,172]. In this regard, studies for the identification of biomarkers that could be integrated into the current histopathological classification [159] for a more granular diagnostic and prognostic stratification of LN patients are needed and form an integral part of the future research agenda in this field.

Among the limitations of this systematic literature review, some aspects need to be underlined as possible sources of bias. Firstly, although not unexpectedly, the biomarker studies deemed eligible for data extraction were characterised by a high degree of heterogeneity in terms of design, patient populations, and definitions of outcomes, which, together with the inevitable inconsistency of laboratory testing across studies, introduces limitations into the generalisability of the findings and makes conclusions hard to draw. For instance, different approaches were followed for the determination of the cut-off values across studies investigating the same molecules, and the diagnosis of LN was not confirmed by a histological assessment with a kidney biopsy in all the studies. Secondly, descriptions of participant characteristics were not sufficiently explicit in all studies. Thirdly, important confounding factors were not always adequately accounted for in the investigations. Detailed information about such limiting factors is presented in Supplementary Tables S2–S4. The overall lack of validation studies in independent cohorts is a major limitation in biomarker research. Efforts should be made to prompt more concerted investigations following centralised approaches. In such efforts, the Lupus Nephritis Trials Network (http://lupusnephritis.org, accessed on 8 August 2022) and other similar initiatives could be instrumental.

Nevertheless, in view of the rapid technological advancements, we foresee a revolution toward the optimised and personalised management of patients with LN in the years to come using a battery of next-generation biomarkers.

## Figures and Tables

**Table 1:** Performances of selected diagnostic biomarkers for LN.

Biomarker	Sample	Comparator	Metrics	References
Autoantibodies				
Anti-C1q (+)	Serum/Plasma	Non-renal SLE	AUC = 0.76; sens.: 74%; spec.: 55%	Gomez-Puerta et al., 2018 [41]
		Non-renal SLE	OR = 4.4	Sjöwall et al., 2018 [50]
		Non-renal SLE	sens.: 63%; spec.: 71%	Birmingham et al., 2016 [51]
		Active non-renal SLE	AUC = 0.64; sens.: 47%; spec.: 83%	Pang et al., 2016 [52]
Anti-dsDNA (+)	Serum/Plasma	Non-renal SLE	AUC = 0.65	Bruschi et al., 2021 [20]*
		Healthy controls	AUC = 0.94	
		Non-renal SLE	OR = 2.1	Hardt et al., 2018 [53]
		Non-renal SLE	AUC = 0.72; sens.: 72%; spec.: 73%; HR = 5.8; HR_adj_ = 2.7	Liu et al., 2021 [54]
		Non-renal SLE	AUC = 0.89; sens.: 100%; spec.: 71%; PPV:44%; NPV: 100%; HR = 1.1	Kwon et al., 2020 [55]
		Active non-renal SLE; Inactive SLE	sens.: 94%; spec.: 40%; PPV: 43%; NPV: 93%	Mok et al., 2016 [56]
		Non-renal SLE	OR = 2.9	Sjöwall et al., 2018 [50]
		Non-renal SLE	OR = 3.3	Barnado et al., 2019 [57]
		Anti-dsDNA-negative SLE	OR = 4.6	
Anti-ENO-1 (+)	Serum/Plasma	Non-renal SLE	AUC = 0.81; sens.: 82%; spec.: 91%	Huang et al., 2019 [18]
		Non-renal SLE	AUC = 0.82	Bruschi et al., 2021 [20] *
		Healthy controls	AUC = 0.94	
PHACTR4 icx (+)	Serum/Plasma	Healthy controls	AUC = 0.99	Tang et al., 2022 [58]
P3H1 icx (+)			AUC = 0.82	
RGS12 icx (+)			AUC = 0.90	
**Complements**				
C3 (low)	Serum/Plasma	Non-renal SLE	HR = 6.4	Liu et al., 2021 [54]
		Non-renal SLE	sens.: 78%; spec.: 92%; PPV: 97%; NPV: 58%; OR = 39	Ishizaki et al., 2015 [59]
		Non-renal SLE	sens.: 74%; spec.: 64%; PPV: 67%; NPV: 71%; OR = 5.0	Martin et al., 2020 [60]
		Active non-renal SLE; Inactive SLE	sens.: 97%; spec.: 32%; PPV: 41%; NPV: 95%	Mok et al., 2016 [56]
C4 (low)	Serum/Plasma	Non-renal SLE	sens.: 70%; spec.: 68%; PPV: 69%; NPV: 70%; OR = 5.1	Martin et al., 2020 [56]
		Non-renal SLE	HR = 5.0	Liu et al., 2021 [54]
**Kidney disease-related markers**				
Albumin to globulin ratio (low)	Urine	Non-renal SLE	AUC = 0.65; sens.: 84%; spec.: 52%; HR = 5.5; HR_adj_ = 7.0	Liu et al., 2021 [54]
Creatinine (**↑**)	Serum/Plasma	Non-renal SLE	AUC = 0.83; sens.: 75%; spec.: 76%; PPV: 86%; NPV: 61%	Yang et al., 2016 [23]
Proteinuria (**↑**) (>500 mg/24 h)	Urine	Non-renal SLE	AUC = 0.99	Jakiela et al., 2018 [61]
Urea (**↑**)	Serum/Plasma	Non-renal SLE	AUC = 0.82; sens.: 60%; spec.: 94%; PPV: 95%; NPV: 55%	Yang et al., 2016 [23]
Uric acid (**↑**)	Serum/Plasma	Non-renal SLE	AUC = 0.86; sens.: 78%; spec.: 79%; PPV: 70%; NPV: 75%	Calich et al., 2018 [22]
		Non-renal SLE	AUC = 0.80; sens.: 75%; spec.: 78%; PPV: 87%; NPV: 62%	Yang et al., 2016 [23]
		Non-renal SLE	AUC = 0.81; sens.: 83%; spec.: 70%; PPV: 74%; NPV: 80%	Hafez et al., 2021 [24]
**Cytokines/chemokines**				
APRIL (**↑**)	Urine	Active non-renal SLE	AUC = 0.78	Phatak et al., 2017 [62]
		Non-renal SLE	sens.: 38%; spec.: 68%	Vincent et al., 2018 [63]
BAFF (**↑**)	Urine	Active non-renal SLE	AUC = 0.83	Phatak et al., 2017 [62]
		Non-renal SLE	sens.: 20%; spec.: 91%	Vincent et al., 2018 [63]
CXCL4 (**↑**)	Urine	Active non-renal SLE	AUC = 0.64; sens.: 63%; spec.: 61%	Mok et al., 2018 [64]
MCP-1 (**↑**)	Urine	Non-renal SLE	AUC = 0.73; sens.: 76%; spec.: 58%	Gómez-Puerta et al., 2018 [41]
		Non-renal SLE	AUC = 0.70	Barbado et al., 2012 [65]
		Non-renal SLE	AUC = 1.00; sens.: 95%; spec.: 93%; PPV: 94%; NPV: 95%	Elsaid et al., 2021 [30]
		Healthy controls	AUC = 0.87	Singh et al., 2012 [66]
TWEAK (**↑**)	Serum/Plasma	Non-renal SLE	AUC = 0.65; sens.: 81%; spec.: 48%; accuracy: 63%; OR = 1.1	Choe et al., 2016 [32]
		Active non-renal SLE	AUC = 0.80; sens.: 80%; spec.: 80%	Mirioglu et al., 2020 [31]
	Urine	Non-renal SLE	AUC = 0.88; sens.:100%; spec.: 67%	Salem et al., 2018 [26]
		Non-renal SLE	AUC = 0.87; sens.: 81%; spec.: 67%	Reyes-Martínez et al., 2018 [27]
		Non-renal SLE	AUC = 1.00; sens.: 100%; spec.: 100%; PPV: 100%; NPV: 100%	Elsaid et al., 2021 [30]
**Cell adhesion molecules**				
ALCAM (**↑**)	Urine	Active non-renal SLE	AUC = 0.75–0.96	Chalmers et al., 2022 [67]
		Healthy controls	AUC = 0.82–0.96	
		Active non-renal SLE	AUC = 0.84	Ding et al., 2020 [68]
		Healthy controls	AUC = 0.93	
VCAM-1 (**↑**)	Urine	Active non-renal SLE	AUC = 0.73–0.92; sens.: 69%; spec.: 66%	Mok et al., 2018 [64]
		Healthy controls	AUC = 0.92	Singh et al., 2012 [66]
**Other proteins**				
Angiostatin (**↑**)	Urine	Active non-renal SLE	AUC = 0.87; sens.: 80%; spec.: 82%	Mok et al., 2018 [64]
		Healthy controls	AUC = 0.95	Wu et al., 2013 [69]
Axl (**↑**)	Serum/Plasma	Active non-renal SLE; Inactive SLE	sens.: 68%; spec.: 77%; PPV: 55%; NPV: 86%	Mok et al., 2016 [56]
HE4 (**↑**)	Serum/Plasma	Non-renal SLE	AUC = 0.88; sens.: 77%; spec.: 91%	Yang et al., 2016 [23]
		Non-renal SLE	AUC = 0.71; sens.: 82%; spec.: 53%; HR = 16.8	Ren et al., 2018 [70]
IGFBP-2 (**↑**)	Serum/Plasma	CKD not LN	AUC = 0.65	Ding et al., 2016 [71]
		Healthy controls	AUC = 0.97	
NGAL (**↑**)	Urine	Active non-renal SLE; Inactive SLE	sens.: 71%; spec.: 90%; PPV: 61%; NPV: 94%	Mok et al., 2016 [56]
		Non-renal SLE	AUC = 0.99; sens.: 98%; spec.: 100%	Li et al., 2019 [42]
		Non-renal SLE	AUC = 0.70; sens.: 67%; spec.: 63%	Gómez-Puerta et al., 2018 [41]
sTNFRII (**↑**)	Serum/Plasma	Active non-renal SLE; Inactive SLE	sens.: 41%; spec.: 81%; PPV: 48%; NPV: 86%	Mok et al., 2016 [56]
TF (**↑**)	Urine	Non-renal SLE	AUC = 0.81	Davies et al., 2021 [72]
		Non-renal SLE	AUC = 0.86	Urrego et al., 2020 [73]
β2-MG (**↑**)	Urine	Non-renal SLE	AUC = 0.85; sens.: 82%; spec.: 90%	Huang et al., 2019 [18]
		Non-renal SLE	OR = 1.1	Choe et al., 2014 [74]
**MicroRNAs**				
miRNA-21 (**↑**)	Serum/Plasma	Non-renal SLE; Inactive LN	AUC = 0.89; OR_adj_ = 3.2	Khoshmirsafa et al., 2019 [45]
		Healthy controls	AUC = 0.91; sens.: 86%; spec.: 63%; PPV: 76%; NPV: 93%	Nakhjavani et al., 2019 [46]
**Microparticles**				
MP-CX3CR1^+^ (**↑**)	Urine	Non-renal SLE	AUC = 0.85; sens.: 63%; spec.: 86%	Burbano et al. [48]
MP-HLADR^+^ (**↑**)			AUC = 0.97; sens.: 85%; spec.: 86%	
MP-HMGB1^+^ (**↑**)			AUC = 0.99–1.00; sens.: 95–100%; spec.: 88%	
**Renal tissue markers**				
Mannose enriched N-glycan expression (GNA reactivity ≥ 50%)	Kidney biopsy	Healthy controls	AUC = 0.83	Alves et al., 2021 [75]

Biomarkers are structured into subgroups (highlighted in bold) based on clinical/functional affinities. ALCAM: activated leukocyte cell adhesion molecule; Anti-dsDNA: anti-double-stranded DNA; Anti-ENO-1: anti-α-enolase 1; APRIL: a proliferation-inducing ligand; AUC: area under the curve; BAFF: B cell activating factor belonging to the TNF ligand superfamily; β2-MG: β_2_-microglobulin; CKD: chronic kidney disease; CXCL4: C-X-C motif chemokine ligand 4; CX3CR1: C-X3-C motif chemokine receptor 1; C3: complement component 3; C4: complement component 4; GNA: galantus nivalis agglutinin reaction; HE4: human epididymis protein 4; HMGB1: high mobility group box 1; HR: hazard ratio; HR_adj_: adjusted hazard ratio; icx: immune complexes; IGFBP-2: insulin-like growth factor binding protein 2; LN: lupus nephritis; MCP-1: monocyte chemoattractant protein 1; miRNA-21: microRNA-21; MP: microparticle; NGAL: neutrophil gelatinase associated lipocalin; NPV: negative predictive value; OR: odds ratio; PHACTR4: phosphatase and actin regulator 4; PPV: positive predictive value; P3H1: prolyl 3-hydroxylase 1; RGS12: regulator of G-protein signalling 12; sens.: sensitivity; SLE: systemic lupus erythematosus; spec.: specificity; sTNFRII: soluble tumour necrosis factor alpha receptor II; TF: transferrin; TWEAK: TNF-like weak inducer of apoptosis; VCAM-1: vascular cell adhesion molecule 1; (+): positivity; **↑**: elevated. * This study evaluated IgG2 subclass antibodies.

**Table 6:** Performances of selected prognostic biomarkers in LN.

Biomarker	Sample	Main Findings	References
Autoantibodies			
ANCAs (+)	Serum/Plasma	Predictive of increased mortality: RR_adj_ = 3.6; HR = 3.3; HR_adj_ = 3.4	Wang et al., 2016 [153]; Wang et al., 2020 [154]
Anti-C1q * (+)	Serum/Plasma	Risk factor for composite outcome (death and doubling of serum creatinine or ESKD) after median follow up of 42 months: HR = 3.9; HR_adj_ = 1.2	Pang et al., 2016 [52]
**Complement**			
C3 (low)	Serum/Plasma	Predictive of renal failure within 20 years: RR_adj_ = 2.0	Petri et al., 2021 [4]
**Kidney disease-related markers**			
Creatinine (**↑**)	Serum/Plasma	Higher baseline levels predictive of ESKD: HR = 2.1	Chen et al., 2019 [169]
		Risk factor for composite outcome after median follow up of 42 months: HR_adj_ = 4.7	Pang et al., 2016 [52]
Proteinuria (**↑**) (>500 mg/24 h)	Urine	Predictive of renal failure within 20 years: RR_adj_ = 2.8	Petri et al., 2021 [4]
		Proteinuric remission indicates good prognosis in patients with diffuse proliferative LN (mean follow up: 157.9 months). RR of composite outcome (sum of mortality and incidence of end stage renal disease) = 0.2	Koo et al., 2016 [150]
**Cell adhesion molecules**			
ALCAM(**↑**) (ALCAM/Cr > 0.18 × 10^−4^) (ALCAM/Cr > 0.17 × 10^−4^)	Urine	High baseline values are predictive of renal function deterioration (decline in eGFR by ≥25%) at the 10-year follow up. AUC = 0.74; sens.: 73%; spec.: 72%; OR = 6.1	Parodis et al., 2020 [99]
VCAM-1 (**↑**) (VCAM1/Cr > 0.32 × 10^−4^) (VCAM1/Cr > 0.24 × 10^−4^)	Urine	High baseline values are predictive of renal function deterioration (decline in eGFR by ≥25%) at the 10-year follow up. AUC = 0.77; sens.: 91; spec.: 76%; OR= 22.9	Parodis et al., 2020 [99]
**Other proteins/soluble molecules**			
Axl (**↑**) (>46.1 ng/mL)	Serum/Plasma	High post treatment values predict good renal outcome (creatinine ≤88.4 μmol/L) over 10 years. AUC = 0.71; sens.: 42%; spec.: 91%; PPV: 80%; NPV: 65%	Parodis et al., 2019 [131]
CD163 (**↑**) (>370 ng/mmol)	Urine	Increased risk for doubling of serum creatinine within 6 (HR = 2.8) and 12 (HR = 3.6) months	Mejia-Vilet et al., 2020 [101]
EGF (**↓**) (EGF/Cr <5.3 ng/mg at flare time)	Urine	Predicts doubling serum creatinine within 2 years. AUC = 0.82; sens.: 81%; spec.: 77%	Mejia-Vilet et al., 2021 [170]
sTNFRII (**↑**) (>7.1 ng/mL)	Serum/Plasma	Higher post treatment levels in CKD≥3 patients compared to CKD1-2 patients. AUC = 0.73; sens.: 73%; spec.: 75%	Parodis et al., 2017 [83]
**Renal tissue markers**			
Arteriolar C4d deposition (+)	Kidney biopsy	Risk factor for poor renal outcome (average follow up time: 55.8 months): HR = 2.1	Ding et al., 2021 [165]
Cellular crescents (+)	Kidney biopsy Kidney biopsy	Predictive of ESKD: HR = 4.4 (cellular crescents) and HR = 5.9 (fibrous crescents)	Chen et al., 2019 [169]
Fibrous crescents (+)			
Glomerular C3 deposition (+)	Kidney biopsy	Positive staining without C1q and C4 deposition (suggestive of alternative pathway-limited activation) is associated with progression of kidney disease (≥50% reduction in eGFR from baseline values or advancement to ESKD) after a mean follow-up of 5.4 years: HR = 4.8; HR_adj_ = 3.5	Kim et al., 2020 [166]
IFTA (+) (≥25% of the surface cortical area)	Kidney biopsy	Moderate/severe IFTA is associated with ESKD (HR_adj_ = 5.2) and death (HR_adj_ = 4.2)	Leatherwood et al., 2019 [164]
Mannose enriched N-glycan expression (GNA reactivity ≥50%)	Kidney biopsy	Increased risk of developing CKD after 1 year: AUC = 0.83; sens.: 67%; spec.: 94%; PPV: 80%; NPV: 87%; OR = 24.3	Alves et al., 2021 [75]
Vascular injury (+) (≥25% subintimal narrowing of the lumen)	Kidney biopsy	Moderate/severe vascular injury is associated with ESKD (HR_adj_ = 2.1)	Leatherwood et al., 2019 [164]

Biomarkers are structured into subgroups (highlighted in bold) based on clinical/functional affinities. ALCAM: activated leukocyte cell adhesion molecule; ANCA: anti-neutrophil cytoplasmic antibody; AUC: area under the curve; CKD: chronic kidney disease; Cr: creatinine; C1q: complement component 1q; C3: complement component 3; C4: complement component 4; C4d: complement component 4d; EGF: epidermal growth factor; eGFR: estimated glomerular filtration rate; ESKD: end stage kidney disease; GNA: galantus nivalis agglutinin reaction; HR: hazard ratio; IFTA: interstitial fibrosis and tubular atrophy; NPV: negative predictive value; OR: odds ratio; PPV: positive predictive value; RR: risk ratio; sens.: sensitivity; spec.: specificity; sTNFRII: soluble tumor necrosis factor alpha receptor II; VCAM-1: vascular cell adhesion molecule 1; (+): positivity; **↑**: increased; **↓**: decreased. * Antibodies against the epitope A08 of C1q.

## Data Availability

No new data were created or analyzed in this study. Data sharing is not applicable to this article.

## Supplementary Materials

The following supporting information can be downloaded at https://www.mdpi.com/article/10.3390/jcm11195759/s1: Figure S1: PRISMA flow diagram for systematic reviews that included searches of databases and registers only. Table S1. Search in Medline. Table S2. Risk of bias assessment of cross-sectional studies. Table S3. Risk of bias assessment of meta-analyses. Table S4. Risk of bias assessment of cohort studies. Table S5. Ethnicity and/or nationality of the populations in the included studies.
