# Supplementary material for: Current Insights on Biomarkers in Lupus Nephritis: A Systematic Review of the Literature
Source: J Clin Med. 2022 Sep 28;11(19):5759. doi: 10.3390/jcm11195759 (PMC9570701; doi:10.3390/jcm11195759)
Supplement: Supplementary file 1 [file jcm-11-05759-s001.zip › jcm-1917751-supplementary-updated/Table S1.pdf]

Table S1. Search strategy and terms used for the identification of relevant studies.

Medline

Interface: Ovid MEDLINE(R) and Epub Ahead of Print, In-Process & Other Non-Indexed Citations and Daily<br><br>Date of Search: 13 June 2022<br><br>Number of hits: X<br><br>Comment: In Ovid, two or more words are automatically searched as phrases; i.e. no quotation marks are needed	Field labels<br><br>• exp/ = exploded MeSH term<br>• / = non exploded MeSH term<br>• .ti,ab,kf. = title, abstract and author keywords<br>• adjx = within x words, regardless of order<br>• * = truncation of word for alternate endings

#	Query	Hits	Comments
**Block 1: Lupus nephritis**			
1	exp Lupus Nephritis/	7,422	
2	lupus.jw.	6,190	Journal dedicated to SLE.
3	(lupus adj3 (auto-immun* or autoimmun* or disseminat* or erythemat* or nephriti* or systemic*)).ti,ab,kf.	72,991	Terms choosen from MeSH (incl entry terms).
4	(SLE and lupus).ti,ab,kf.	33,502	Both SLE and lupus can be interpreted otherwise. Thus, combination was chosen.
5	or/1-4	75,764	
**Block 2: Biomarkers**			
6	exp Biomarkers/	849,647	Includes the following narrower terms: “Antibodies, Antineutrophil Cytoplasmic”, “Antigens, Differentiation”, “Biomarkers, Pharmacological”, “Biomarkers, Tumor”, “Chorionic Gonadotropin, beta Subunit, Human”, “Environmental Biomarkers”, “Fibrinopeptide A”, “Genetic Markers”, “Oligoclonal Bands”
7	(biomarker* or marker*).jw.	21,480	
8	(biomarker* or biological marker* or diagnostic marker*).ti,ab,kf.	377,907	Biological and diagnostic causes too much noise with ADJx.
9	((immunol* or metabol* or molecular* or novel) adj3 marker*).ti,ab,kf.	60,010	ADJ3 reasonable, since more than two words in between gives much noise.
10	((blood or plasma or sera or serologic* or serum or urinary or urine or kidney or renal or tissue or biopsy) adj3 marker*).ti,ab,kf.	51,125	Terms for where markers may be found. See #9.
11	((antibod* or antigen* or autoantibod* or cytokine* or glycoprotein* or immunoglob* or lectin* or lipoprotein* or peptide* or protein* or RNA) adj3 marker*).ti,ab,kf.	35,696	Terms for substances commonly occurring as biomarkers. See #9.
12	or/6-11	1,152,708	

13	exp Peptides/	2,701,379	MeSH-terms for substances occurring as biomarkers, to be combined with free-text word marker*.<br><br>Terms included if giving > 10 hits in combination with block 1 and MeSH-term Biomarkers/.
14	exp Blood Proteins/	1,719,977	
15	exp Membrane Proteins/	2,068,907	
16	exp Carrier Proteins/	1,266,955	
17	exp Glycoproteins/	896,093	
18	exp Lipoproteins/	156,218	
19	exp DNA-Binding Proteins/	612,390	
20	exp Cytoskeletal Proteins/	306,994	
21	exp "Receptors, Cytoplasmic and Nuclear"/	174,510	
22	exp Lectins/	117,563	
23	or/13-22	6,047,259	
24	marker*.ti,ab,kf.	865,970	
25	23 AND 24	361,443	
26	12 or 25	1,326,012	
**Block 3: Study types for exclusion**			
27	exp Animals/ not Humans/	5,016,581	Based on a filter from McGill University.
28	exp Models, Animal/ not Humans/	432,376	
29	exp Animal Experimentation/ not Humans/	5,818	
30	(animal or animals or canine or dog or dogs or feline or hamster or hamsters or lamb or lambs or mice or monkey or monkeys or mouse or murine or pig or pigs or piglet* or porcine or primate or primates or rabbit or rabbits or rat or rats or rodent* or sheep or sheeps or veterinar*).ti,kf,jw.	2,582,359	
31	or/27-30	5,528,666	
32	case reports.pt.	2,273,684	
33	case.jw.	121,799	
34	(case adj (report* or series or stud*)).ti,kf.	409,604	ADJ1 (or more) include case-control studies.
35	or/33-34	500,454	
36	31 OR 35	6,022,365	
**Result: Biomarkers in lupus nephritis**			
37	(5 and 26) not 36	7,550	
38	limit 37 to (yr="2012 -Current" and "all adult (19 plus years)" and english)	1,994	Limits based on exclusion criteria.
