# Supplementary material for: Current Insights on Biomarkers in Lupus Nephritis: A Systematic Review of the Literature
Source: J Clin Med. 2022 Sep 28;11(19):5759. doi: 10.3390/jcm11195759 (PMC9570701; doi:10.3390/jcm11195759)
Supplement: Supplementary file 1 [file jcm-11-05759-s001.zip › jcm-1917751-supplementary-updated/Table S2.pdf]

Table S2. Risk of bias assessment of cross-sectional studies*.

Author, year	Were the criteria for inclusion in the sample clearly defined?	Were the study subjects and the setting described in detail?	Was the exposure measured in a valid and reliable way?	Were objective, standard criteria used for measurement of the condition?	Were confounding factors identified?	Were strategies to deal with confounding factors stated?	Were the outcomes measured in a valid and reliable way?	Was appropriate statistical analysis used?
Alharazy et al., 2013 [1]	Yes	Yes	Not applicable	Yes	Unclear	Unclear	Not applicable	Yes
Alves et al., 2021[2]	No	No	Not applicable	Yes	Yes	Unclear	Not applicable	Yes
Barbado et al., 2012 [3]	Yes	No	Not applicable	Yes	No	No	Not applicable	Yes
Barnado et al., 2019 [4]	Yes	Yes	Not applicable	Yes	Yes	Yes	Not applicable	Yes
Birmingham et al.m 2016 [5]	Yes	Yes	Not applicable	Yes	No	No	Not applicable	Yes
Bock et al., 2015 [6]	Yes	Yes	Not applicable	Yes	No	No	Not applicable	Yes
Bruschi et al., 2021 [7]	Yes	Yes	Not applicable	Yes	No	No	Not applicable	Yes
Burbano et al., 2019 [8]	Yes	No	Not applicable	Yes	Yes	Unclear	Not applicable	Yes
Calich et al., 2018 [9]	Yes	Yes	Not applicable	Yes	Yes	Yes	Not applicable	Yes
Chalmers et al., 2022 [10]	Yes	Yes	Not applicable	Yes	Yes	Yes	Not applicable	Yes
Choe et al., 2014 [11]	Yes	No	Not applicable	Yes	Yes	Yes	Not applicable	Yes
Choe et al., 2016 [12]	Yes	No	Not applicable	Yes	Yes	Yes	Not applicable	Yes
Ding et al., 2016 [13]	Yes	No	No applicable	Yes	Unclear	No	Not applicable	Yes
Ding et al., 2020 [14]	Yes	Yes	Not applicable	Yes	No	No	Not applicable	Yes
Dolff et al., 2013 [15]	Yes	No	Not applicable	Yes	No	No	Not applicable	Yes
Elsaid et al., 2021 [16]	Yes	No	Not applicable	Yes	No	No	Not applicable	Yes
Endo et al., 2016 [17]	No	No	Not applicable	Yes	No	No	Not applicable	Yes
Enghard et al., 2014 [18]	No	Yes	Not applicable	Yes	No	No	Not applicable	Yes
Fava et al., 2022 [19]	Yes	Yes	Not applicable	Yes	Yes	Yes	Not applicable	Yes
Go et al., 2018 [20]	Yes	No	Not applicable	Yes	Yes	Yes	Not applicable	Yes

Gomez-Puerta et al., 2018 [21]	Yes	Yes	Not applicable	Yes	No	No	Not applicable	Yes
Hafez et al., 2021 [22]	No	No	Not applicable	Yes	No	No	Not applicable	Yes
Hardt et al., 2018 [23]	Yes	No	Not applicable	Yes	Yes	Yes	Not applicable	Yes
Howe et al., 2012 (24)	No	No	Not applicable	Yes	No	No	Not applicable	Yes
Huang et al., 2019 [24]	No	No	Not applicable	Yes	No	No	Not applicable	Yes
Hutcheson et al., 2015 [25]	Yes	Yes	Not applicable	Yes	No	No	Not applicable	Yes
Ichinose et al., 2018 [26]	No	Yes	Not applicable	Yes	Yes	Yes	Not applicable	Yes
Ishizaki et al., 2015 [27]	Yes	Yes	Not applicable	Yes	Yes	Yes	Not applicable	Yes
Jakiela et al., 2018 [28]	Yes	Yes	Not applicable	Yes	No	No	Not applicable	Yes
Khoshmirsafa et al., 2015 [29]	No	Yes	Not applicable	Yes	Yes	Yes	Not applicable	Yes
Khianmehr et al., 2021 [30]	No	Yes	Not applicable	Yes	No	No	Not applicable	Yes
Kim et al., 2015 [31]	Yes	Yes	Not applicable	Yes	No	No	Not applicable	Yes
Koo et al., 2016 [32]	Yes	Yes	Not applicable	Yes	Yes	Yes	Yes	Yes
Mejia-Vilet et al., 2020 [33]	No	Yes	Not applicable	Yes	No	No	Not applicable	Yes
Menke et al., 2015 [34]	No	Yes	Not applicable	Yes	No	No	Not applicable	Yes
Mirioglu et al., 2020 [35]	No	No	Not applicable	Yes	No	No	Not applicable	Yes
Mok et al., 2016 [36]	No	No	Not applicable	Yes	Yes	Yes	Not applicable	Yes
Mok et al., 2018 [37]	Yes	No	Not applicable	Yes	No	No	Not applicable	Yes
Nakhjavani et al., 2019 [38]	Yes	No	Not applicable	Yes	No	No	Not applicable	Yes
Nordin et al., 2019 [39]	No	No	Not applicable	Yes	No	No	Not applicable	Yes
Pang et al., 2016 [40]	No	Yes	Not applicable	Yes	Yes	Yes	Not applicable	Yes
Qin et al., 2019 [41]	Yes	Yes	Not applicable	Yes	Yes	Yes	Not applicable	Yes
Reyes-Martinez et al., 2018 [42]	Yes	No	Not applicable	Yes	No	No	Not applicable	Yes

Rosa et al., 2012 [43]	No	No	Not applicable	Yes	No	No	Not applicable	Yes
Salem et al., 2018 [44]	Yes	No	Not applicable	Yes	No	No	Not applicable	Yes
Selvaraja et al., 2019 [45]	No	No	Not applicable	Yes	Yes	Yes	Not applicable	Yes
Singh et al., 2012 [46]	Yes	Yes	Not applicable	Yes	Yes	Yes	Not applicable	Yes
Sjowall et al., 2018 [47]	No	Yes	Not applicable	Yes	Yes	Yes	Not applicable	Yes
Smith et al., 2019 [48]	Yes	Yes	Not applicable	Yes	Yes	Yes	Not applicable	Yes
Soliman et al., 2017 [49]	Yes	Yes	Not applicable	Yes	No	No	Not applicable	Yes
Stanley et al., 2019 [50]	No	No	Not applicable	Yes	No	No	Not applicable	Yes
Stanley et al., 2020 [51]	Yes	Yes	Not applicable	Yes	Yes	Yes	Not applicable	Yes
Tang et al., 2022 [52]	No	Yes	Not applicable	Yes	No	No	Not applicable	Yes
Urrego et al., 2020 [53]	No	Yes	Not applicable	Yes	Yes	Yes	Not applicable	Yes
Urrego-Callejas et al., 2021	Yes	Yes	Not applicable	Yes	No	No	Not applicable	No
Vanarsa et al., 2020 [54]	No	No	Not applicable	Yes	Yes	Yes	Not applicable	Yes
Wang et al., 2018 [55]	No	Yes	Not applicable	Yes	No	No	Not applicable	Yes
Wantanasiri et al., 2016 [56]	Yes	Yes	Not applicable	Yes	No	No	Not applicable	Yes
Wu et al., 2013 [57]	No	Yes	Not applicable	Yes	No	No	Not applicable	Yes
Wu et al., 2016 [58]	No	Yes	Not applicable	Yes	No	No	Not applicable	Yes
Wu et al., 2016 [59]	Yes	Yes	Not applicable	Yes	Yes	Yes	Not applicable	Yes
Wu et al., 2016 [60]	No	Yes	Not applicable	Yes	No	No	Not applicable	Yes
Yang et al., 2016 [61]	No	Yes	Not applicable	Yes	No	No	Not applicable	Yes
Yu et al., 2021 [62]	Yes	Yes	Not applicable	Yes	No	No	Not applicable	Yes
Zhang et al., 2020 [63]	No	Yes	Not applicable	Yes	Yes	Yes	Not applicable	Yes

* Assessed by Joanna Briggs Institute (JBI) Critical Appraisal Checklist for Analytical Cross Sectional Studies [64].

References

1. Alharazy, S.M.; Kong, N.C.T.; Mohd, M.; Shah, S.A.; Abdul Gafor, A.H.; Ba'in, A. The role of urinary neutrophil gelatinase-associated lipocalin in lupus nephritis. *Clinica chimica acta; international journal of clinical chemistry* **2013**, *425*, 163-168.
2. Alves, I.; Santos-Pereira, B.; Dalebout, H.; Santos, S.; Vicente, M.M.; Campar, A.; Thepaut, M.; Fieschi, F.; Strahl, S.; Boyaval, F.; et al. Protein Mannosylation as a Diagnostic and Prognostic Biomarker of Lupus Nephritis: An Unusual Glycan Neoepitope in Systemic Lupus Erythematosus. *Arthritis & rheumatology (Hoboken, N.J.)* **2021**, *73*, 2069-2077.
3. Barbado, J.; Martin, D.; Vega, L.; Almansa, R.; Goncalves, L.; Nocito, M.; Jimeno, A.; Ortiz de Lejarazu, R.; Bermejo-Martin, J.F. MCP-1 in urine as biomarker of disease activity in Systemic Lupus Erythematosus. *Cytokine* **2012**, *60*, 583-586.
4. Barnado, A.; Carroll, R.J.; Casey, C.; Wheless, L.; Denny, J.C.; Crofford, L.J. Phenome-wide association study identifies dsDNA as a driver of major organ involvement in systemic lupus erythematosus. *Lupus* **2019**, *28*, 66-76, doi:https://dx.doi.org/10.1177/0961203318815577.
5. Mok, C.C.; Birmingham, D.J.; Ho, L.Y.; Hebert, L.A.; Song, H.; Rovin, B.H. Vitamin D deficiency as marker for disease activity and damage in systemic lupus erythematosus: a comparison with anti-dsDNA and anti-C1q. *Lupus* **2012**, *21*, 36-42, doi:https://dx.doi.org/10.1177/0961203311422094.
6. Bock, M.; Heijnen, I.; Trendelenburg, M. Anti-C1q antibodies as a follow-up marker in SLE patients. *PloS one* **2015**, *10*, e0123572.
7. Bruschi, M.; Moroni, G.; Sinico, R.A.; Franceschini, F.; Fredi, M.; Vaglio, A.; Cavagna, L.; Petretto, A.; Pratesi, F.; Migliorini, P.; et al. Serum IgG2 antibody multicomposition in systemic lupus erythematosus and lupus nephritis (Part 1): cross-sectional analysis. *Rheumatology (Oxford, England)* **2021**, *60*, 3176-3188.
8. Burbano, C.; Gomez-Puerta, J.A.; Munoz-Vahos, C.; Vanegas-Garcia, A.; Rojas, M.; Vasquez, G.; Castano, D. HMGB1+ microparticles present in urine are hallmarks of nephritis in patients with systemic lupus erythematosus. *European journal of immunology* **2019**, *49*, 323-335.
9. Calich, A.L.; Borba, E.F.; Ugolini-Lopes, M.R.; da Rocha, L.F.; Bonfa, E.; Fuller, R. Serum uric acid levels are associated with lupus nephritis in patients with normal renal function. *Clinical rheumatology* **2018**, *37*, 1223-1228.
10. Chalmers, S.A.; Ayilam Ramachandran, R.; Garcia, S.J.; Der, E.; Herlitz, L.; Ampudia, J.; Chu, D.; Jordan, N.; Zhang, T.; Parodis, I.; et al. The CD6/ALCAM pathway promotes lupus nephritis via T cell-mediated responses. *J Clin Invest* **2022**, *132*, doi:10.1172/jci147334.
11. Choe, J.Y.; Park, S.H.; Kim, S.K. Urine beta2-microglobulin is associated with clinical disease activity and renal involvement in female patients with systemic lupus erythematosus. *Lupus* **2014**, *23*, 1486-1493, doi:https://dx.doi.org/10.1177/0961203314547797.
12. Choe, J.-Y.; Kim, S.-K. Serum TWEAK as a biomarker for disease activity of systemic lupus erythematosus. *Inflammation research : official journal of the European Histamine Research Society ... [et al.]* **2016**, *65*, 479-488.
13. Ding, H.; Kharboutli, M.; Saxena, R.; Wu, T. Insulin-like growth factor binding protein-2 as a novel biomarker for disease activity and renal pathology changes in lupus nephritis. *Clinical and experimental immunology* **2016**, *184*, 11-18.
14. Ding, H.; Lin, C.; Cai, J.; Guo, Q.; Dai, M.; Mohan, C.; Shen, N. Urinary activated leukocyte cell adhesion molecule as a novel biomarker of lupus nephritis histology. *Arthritis Res Ther* **2020**, *22*, 122, doi:10.1186/s13075-020-02209-9.
15. Dolff, S.; Abdulahad, W.H.; Arends, S.; van Dijk, M.C.R.F.; Limburg, P.C.; Kallenberg, C.G.M.; Bijl, M. Urinary CD8+ T-cell counts discriminate between active and inactive lupus nephritis. *Arthritis research & therapy* **2013**, *15*, R36.

16. Elsaid, D.S.; Abdel Noor, R.A.; Shalaby, K.A.; Haroun, R.A.-H. Urinary Tumor Necrosis Factor-Like Weak Inducer of Apoptosis (uTWEAK) and Urinary Monocyte Chemo-attractant Protein-1 (uMCP-1): Promising Biomarkers of Lupus Nephritis Activity? *Saudi journal of kidney diseases and transplantation : an official publication of the Saudi Center for Organ Transplantation, Saudi Arabia* **2021**, *32*, 19-29.
17. Endo, N.; Tsuboi, N.; Furuhashi, K.; Shi, Y.; Du, Q.; Abe, T.; Hori, M.; Imaizumi, T.; Kim, H.; Katsuno, T.; et al. Urinary soluble CD163 level reflects glomerular inflammation in human lupus nephritis. *Nephrology, dialysis, transplantation : official publication of the European Dialysis and Transplant Association - European Renal Association* **2016**, *31*, 2023-2033.
18. Enghard, P.; Rieder, C.; Kopetschke, K.; Klocke, J.R.; Undeutsch, R.; Biesen, R.; Dragun, D.; Gollasch, M.; Schneider, U.; Aupperle, K.; et al. Urinary CD4 T cells identify SLE patients with proliferative lupus nephritis and can be used to monitor treatment response. *Annals of the rheumatic diseases* **2014**, *73*, 277-283.
19. Fava, A.; Rao, D.A.; Mohan, C.; Zhang, T.; Rosenberg, A.; Fenaroli, P.; Belmont, H.M.; Izmirly, P.; Clancy, R.; Trujillo, J.M.; et al. Urine Proteomics and Renal Single-Cell Transcriptomics Implicate Interleukin-16 in Lupus Nephritis. *Arthritis Rheumatol* **2022**, *74*, 829-839, doi:10.1002/art.42023.
20. Go, D.J.; Lee, J.Y.; Kang, M.J.; Lee, E.Y.; Lee, E.B.; Yi, E.C.; Song, Y.W. Urinary vitamin D-binding protein, a novel biomarker for lupus nephritis, predicts the development of proteinuric flare. *Lupus* **2018**, *27*, 1600-1615, doi:https://dx.doi.org/10.1177/0961203318778774.
21. Gomez-Puerta, J.A.; Ortiz-Reyes, B.; Urrego, T.; Vanegas-Garcia, A.L.; Munoz, C.H.; Gonzalez, L.A.; Cervera, R.; Vasquez, G. Urinary neutrophil gelatinase-associated lipocalin and monocyte chemoattractant protein 1 as biomarkers for lupus nephritis in Colombian SLE patients. *Lupus* **2018**, *27*, 637-646, doi:https://dx.doi.org/10.1177/0961203317738226.
22. Hafez, E.A.; Hassan, S.A.E.-M.; Teama, M.A.M.; Badr, F.M. Serum uric acid as a predictor for nephritis in Egyptian patients with systemic lupus erythematosus. *Lupus* **2021**, *30*, 378-384.
23. Hardt, U.; Larsson, A.; Gunnarsson, I.; Clancy, R.M.; Petri, M.; Buyon, J.P.; Silverman, G.J.; Svenungsson, E.; Gronwall, C. Autoimmune reactivity to malondialdehyde adducts in systemic lupus erythematosus is associated with disease activity and nephritis. *Arthritis research & therapy* **2018**, *20*, 36.
24. Huang, Y.; Chen, L.; Chen, K.; Huang, F.; Feng, Y.; Xu, Z.; Wang, W. Anti-alpha-enolase antibody combined with beta2 microglobulin evaluated the incidence of nephritis in systemic lupus erythematosus patients. *Lupus* **2019**, *28*, 365-370, doi:https://dx.doi.org/10.1177/0961203319828822.
25. Hutcheson, J.; Ye, Y.; Han, J.; Arriens, C.; Saxena, R.; Li, Q.Z.; Mohan, C.; Wu, T. Resistin as a potential marker of renal disease in lupus nephritis. *Clinical and experimental immunology* **2015**, *179*, 435-443.
26. Ichinose, K.; Kitamura, M.; Sato, S.; Fujikawa, K.; Horai, Y.; Matsuoka, N.; Tsuboi, M.; Nonaka, F.; Shimizu, T.; Fukui, S.; et al. Podocyte foot process width is a prediction marker for complete renal response at 6 and 12months after induction therapy in lupus nephritis. *Clinical immunology (Orlando, Fla.)* **2018**, *197*, 161-168.
27. Ishizaki, J.; Saito, K.; Nawata, M.; Mizuno, Y.; Tokunaga, M.; Sawamukai, N.; Tamura, M.; Hirata, S.; Yamaoka, K.; Hasegawa, H.; et al. Low complements and high titre of anti-Sm antibody as predictors of histopathologically proven silent lupus nephritis without abnormal urinalysis in patients with systemic lupus erythematosus. *Rheumatology (Oxford, England)* **2015**, *54*, 405-412.
28. Jakiela, B.; Kosalka, J.; Plutecka, H.; Wegrzyn, A.S.; Bazan-Socha, S.; Sanak, M.; Musial, J. Urinary cytokines and mRNA expression as biomarkers of disease activity in lupus nephritis. *Lupus* **2018**, *27*, 1259-1270, doi:https://dx.doi.org/10.1177/0961203318770006.

29. Khoshmirsafa, M.; Kianmehr, N.; Falak, R.; Mowla, S.J.; Seif, F.; Mirzaei, B.; Valizadeh, M.; Shekarabi, M. Elevated expression of miR-21 and miR-155 in peripheral blood mononuclear cells as potential biomarkers for lupus nephritis. *International journal of rheumatic diseases* **2019**, *22*, 458-467.
30. Kianmehr, N.; Khoshmirsafa, M.; Shekarabi, M.; Falak, R.; Haghighi, A.; Masoodian, M.; Seif, F.; Omidi, F.; Shirani, F.; Dadfar, N. High frequency of concurrent anti-C1q and anti-dsDNA but not anti-C3b antibodies in patients with Lupus Nephritis. *Journal of immunoassay & immunochemistry* **2021**, *42*, 406-423.
31. Kim, K.-J.; Kim, J.-Y.; Baek, I.-W.; Kim, W.-U.; Cho, C.-S. Elevated serum levels of syndecan-1 are associated with renal involvement in patients with systemic lupus erythematosus. *The Journal of rheumatology* **2015**, *42*, 202-209.
32. Koo, H.S.; Kim, S.; Chin, H.J. Remission of proteinuria indicates good prognosis in patients with diffuse proliferative lupus nephritis. *Lupus* **2016**, *25*, 3-11, doi:https://dx.doi.org/10.1177/0961203315595130.
33. Mejia-Vilet, J.M.; Zhang, X.L.; Cruz, C.; Cano-Verduzco, M.L.; Shapiro, J.P.; Nagaraja, H.N.; Morales-Buenrostro, L.E.; Rovin, B.H. Urinary Soluble CD163: a Novel Noninvasive Biomarker of Activity for Lupus Nephritis. *Journal of the American Society of Nephrology : JASN* **2020**, *31*, 1335-1347.
34. Menke, J.; Amann, K.; Cavagna, L.; Blettner, M.; Weinmann, A.; Schwarting, A.; Kelley, V.R. Colony-stimulating factor-1: a potential biomarker for lupus nephritis. *Journal of the American Society of Nephrology : JASN* **2015**, *26*, 379-389.
35. Mirioglu, S.; Cinar, S.; Yazici, H.; Ozluk, Y.; Kilicaslan, I.; Gul, A.; Ocal, L.; Inanc, M.; Artim-Esen, B. Serum and urine TNF-like weak inducer of apoptosis, monocyte chemoattractant protein-1 and neutrophil gelatinase-associated lipocalin as biomarkers of disease activity in patients with systemic lupus erythematosus. *Lupus* **2020**, *29*, 379-388, doi:https://dx.doi.org/10.1177/0961203320904997.
36. Mok, C.C.; Ding, H.H.; Kharboutli, M.; Mohan, C. Axl, Ferritin, Insulin-Like Growth Factor Binding Protein 2, and Tumor Necrosis Factor Receptor Type II as Biomarkers in Systemic Lupus Erythematosus. *Arthritis care & research* **2016**, *68*, 1303-1309.
37. Mok, C.C.; Soliman, S.; Ho, L.Y.; Mohamed, F.A.; Mohamed, F.I.; Mohan, C. Urinary angiostatin, CXCL4 and VCAM-1 as biomarkers of lupus nephritis. *Arthritis research & therapy* **2018**, *20*, 6.
38. Nakhjavani, M.; Etemadi, J.; Pourlak, T.; Mirhosaini, Z.; Zununi Vahed, S.; Abediazar, S. Plasma levels of miR-21, miR-150, miR-423 in patients with lupus nephritis. *Iranian journal of kidney diseases* **2019**, *13*, 198-206.
39. Nordin, F.; Shaharir, S.S.; Abdul Wahab, A.; Mustafar, R.; Abdul Gafor, A.H.; Mohamed Said, M.S.; Rajalingham, S.; Shah, S.A. Serum and urine interleukin-17A levels as biomarkers of disease activity in systemic lupus erythematosus. *International journal of rheumatic diseases* **2019**, *22*, 1419-1426.
40. Pang, Y.; Tan, Y.; Li, Y.; Zhang, J.; Guo, Y.; Guo, Z.; Zhang, C.; Yu, F.; Zhao, M.-H. Serum A08 C1q antibodies are associated with disease activity and prognosis in Chinese patients with lupus nephritis. *Kidney international* **2016**, *90*, 1357-1367.
41. Qin, L.; Stanley, S.; Ding, H.; Zhang, T.; Truong, V.T.T.; Celhar, T.; Fairhurst, A.-M.; Pedroza, C.; Petri, M.; Saxena, R.; et al. Urinary pro-thrombotic, anti-thrombotic, and fibrinolytic molecules as biomarkers of lupus nephritis. *Arthritis research & therapy* **2019**, *21*, 176.
42. Reyes-Martinez, F.; Perez-Navarro, M.; Rodriguez-Matias, A.; Soto-Abraham, V.; Gutierrez-Reyes, G.; Medina-Avila, Z.; Valdez-Ortiz, R. Assessment of urinary TWEAK levels in Mexican patients with untreated lupus nephritis: An exploratory study. *Nefrologia* **2018**, *38*, 152-160.
43. Rosa, R.F.; Takei, K.; Araujo, N.C.; Loduca, S.M.A.; Szajubok, J.C.M.; Chahade, W.H. Monocyte chemoattractant-1 as a urinary biomarker for the diagnosis of activity of lupus nephritis in Brazilian patients. *The Journal of rheumatology* **2012**, *39*, 1948-1954.

44. Salem, M.N.; Taha, H.A.; Abd El-Fattah El-Feqi, M.; Eesa, N.N.; Mohamed, R.A. Urinary TNF-like weak inducer of apoptosis (TWEAK) as a biomarker of lupus nephritis. *TNF-ahnlicher schwacher Induktor von Apoptose (TWEAK) im Urin als Biomarker einer Lupusnephritis.* **2018**, *77*, 71-77.
45. Selvaraja, M.; Abdullah, M.; Arip, M.; Chin, V.K.; Shah, A.; Amin Nordin, S. Elevated interleukin-25 and its association to Th2 cytokines in systemic lupus erythematosus with lupus nephritis. *PloS one* **2019**, *14*, e0224707.
46. Singh, S.; Wu, T.; Xie, C.; Vanarsa, K.; Han, J.; Mahajan, T.; Oei, H.B.; Ahn, C.; Zhou, X.J.; Putterman, C.; et al. Urine VCAM-1 as a marker of renal pathology activity index in lupus nephritis. *Arthritis research & therapy* **2012**, *14*, R164.
47. Sjowall, C.; Bentow, C.; Aure, M.A.; Mahler, M. Two-Parametric Immunological Score Development for Assessing Renal Involvement and Disease Activity in Systemic Lupus Erythematosus. *Journal of immunology research* **2018**, *2018*, 1294680.
48. Smith, M.A.; Henault, J.; Karnell, J.L.; Parker, M.L.; Riggs, J.M.; Sinibaldi, D.; Taylor, D.K.; Ettinger, R.; Grant, E.P.; Sanjuan, M.A.; et al. SLE Plasma Profiling Identifies Unique Signatures of Lupus Nephritis and Discoid Lupus. *Scientific reports* **2019**, *9*, 14433.
49. Soliman, S.; Mohamed, F.A.; Ismail, F.M.; Stanley, S.; Saxena, R.; Mohan, C. Urine angiostatin and VCAM-1 surpass conventional metrics in predicting elevated renal pathology activity indices in lupus nephritis. *International journal of rheumatic diseases* **2017**, *20*, 1714-1727.
50. Stanley, S.; Mok, C.C.; Vanarsa, K.; Habazi, D.; Li, J.; Pedroza, C.; Saxena, R.; Mohan, C. Identification of Low-Abundance Urinary Biomarkers in Lupus Nephritis Using Electrochemiluminescence Immunoassays. *Arthritis Rheumatol* **2019**, *71*, 744-755, doi:10.1002/art.40813.
51. Stanley, S.; Vanarsa, K.; Soliman, S.; Habazi, D.; Pedroza, C.; Gidley, G.; Zhang, T.; Mohan, S.; Der, E.; Suryawanshi, H.; et al. Comprehensive aptamer-based screening identifies a spectrum of urinary biomarkers of lupus nephritis across ethnicities. *Nature communications* **2020**, *11*, 2197.
52. Tang, C.; Fang, M.; Tan, G.; Zhang, S.; Yang, B.; Li, Y.; Zhang, T.; Saxena, R.; Mohan, C.; Wu, T. Discovery of Novel Circulating Immune Complexes in Lupus Nephritis Using Immunoproteomics. *Front Immunol* **2022**, *13*, 850015, doi:10.3389/fimmu.2022.850015.
53. Urrego, T.; Ortiz-Reyes, B.; Vanegas-Garcia, A.L.; Munoz, C.H.; Gonzalez, L.A.; Vasquez, G.; Gomez-Puerta, J.A. Utility of urinary transferrin and ceruloplasmin in patients with systemic lupus erythematosus for differentiating patients with lupus nephritis. *Transferrina y ceruloplasmina en orina de pacientes con lupus eritematoso sistemico. Son utiles para diferenciar pacientes con nefritis lupica?* **2020**, *16*, 17-23.
54. Vanarsa, K.; Soomro, S.; Zhang, T.; Strachan, B.; Pedroza, C.; Nidhi, M.; Cicalese, P.; Gidley, C.; Dasari, S.; Mohan, S.; et al. Quantitative planar array screen of 1000 proteins uncovers novel urinary protein biomarkers of lupus nephritis. *Ann Rheum Dis* **2020**, *79*, 1349-1361, doi:10.1136/annrheumdis-2019-216312.
55. Wang, Y.; Tao, Y.; Liu, Y.; Zhao, Y.; Song, C.; Zhou, B.; Wang, T.; Gao, L.; Zhang, L.; Hu, H. Rapid detection of urinary soluble intercellular adhesion molecule-1 for determination of lupus nephritis activity. *Medicine* **2018**, *97*, e11287.
56. Wantanasiri, P.; Satirapoj, B.; Charoenpitakchai, M.; Aramwit, P. Periostin: a novel tissue biomarker correlates with chronicity index and renal function in lupus nephritis patients. *Lupus* **2015**, *24*, 835-845, doi:https://dx.doi.org/10.1177/0961203314566634.
57. Wu, T.; Du, Y.; Han, J.; Singh, S.; Xie, C.; Guo, Y.; Zhou, X.J.; Ahn, C.; Saxena, R.; Mohan, C. Urinary angiostatin--a novel putative marker of renal pathology chronicity in lupus nephritis. *Molecular & cellular proteomics : MCP* **2013**, *12*, 1170-1179.
58. Wu, J.; Wei, L.; Wang, W.; Zhang, X.; Chen, L.; Lin, C. Diagnostic value of progranulin in patients with lupus nephritis and its correlation with disease activity. *Rheumatology international* **2016**, *36*, 759-767.

59. Wu, T.; Ding, H.; Han, J.; Arriens, C.; Wei, C.; Han, W.; Pedroza, C.; Jiang, S.; Anolik, J.; Petri, M.; et al. Antibody-Array-Based Proteomic Screening of Serum Markers in Systemic Lupus Erythematosus: A Discovery Study. *Journal of proteome research* **2016**, *15*, 2102-2114.
60. Wu, T.; Xie, C.; Han, J.; Ye, Y.; Singh, S.; Zhou, J.; Li, Y.; Ding, H.; Li, Q.-z.; Zhou, X.; et al. Insulin-Like Growth Factor Binding Protein-4 as a Marker of Chronic Lupus Nephritis. *PloS one* **2016**, *11*, e0151491.
61. Yang, Z.; Zhang, Z.; Qin, B.; Wu, P.; Zhong, R.; Zhou, L.; Liang, Y. Human Epididymis Protein 4: A Novel Biomarker for Lupus Nephritis and Chronic Kidney Disease in Systemic Lupus Erythematosus. *Journal of clinical laboratory analysis* **2016**, *30*, 897-904.
62. Yu, K.Y.; Yung, S.; Chau, M.K.; Tang, C.S.; Yap, D.Y.; Tang, A.H.; Ying, S.K.; Lee, C.K.; Chan, T.M. Clinico-pathological associations of serum VCAM-1 and ICAM-1 levels in patients with lupus nephritis. *Lupus* **2021**, *30*, 1039-1050.
63. Zhang, T.; Li, H.; Vanarsa, K.; Gidley, G.; Mok, C.C.; Petri, M.; Saxena, R.; Mohan, C. Association of Urine sCD163 With Proliferative Lupus Nephritis, Fibrinoid Necrosis, Cellular Crescents and Intrarenal M2 Macrophages. *Frontiers in immunology* **2020**, *11*, 671.
64. JBI. Critical Appraisal Tools. Available online: https://jbi.global/critical-appraisal-tools. (accessed on 4 July 2022).
