# Supplementary material for: Current Insights on Biomarkers in Lupus Nephritis: A Systematic Review of the Literature
Source: J Clin Med. 2022 Sep 28;11(19):5759. doi: 10.3390/jcm11195759 (PMC9570701; doi:10.3390/jcm11195759)
Supplement: Supplementary file 1 [file jcm-11-05759-s001.zip › jcm-1917751-supplementary-updated/Table S3.pdf]

Table S3. Risk of bias assessment of meta-analyses*.

Author, year	Is the review question clearly and explicitly stated?	Were the inclusion criteria appropriate for the review question?	Was the search strategy appropriate?	Were the sources and resources used to search for studies adequate?	Were the criteria for appraising studies appropriate?	Was critical appraisal conducted by two or more reviewers independently?	Were there methods to minimize errors in data extraction?	Were the methods used to combine studies appropriate?	Was the likelihood of publication bias assessed?	Were recommendations for policy and/or practice supported by the reported data?	Were the specific directives for new research appropriate?
Xia et al., 2020 (1)	Yes	Yes	Yes	Yes	Yes	Yes	Unclear	Yes	Yes	Yes	Yes
Zhang et al., 2020 (2)	Yes	Yes	Yes	Yes	Yes	Unclear	Unclear	Yes	Unclear	Yes	Yes

* Assessed by Joanna Briggs Institute (JBI) Critical Appraisal Checklist for Systematic Reviews and Research Syntheses (3).

References

1. Xia Y-R, Li Q-R, Wang J-P, Guo H-S, Bao Y-Q, Mao Y-M, Wu J, Pan H-F, Ye D-Q. Diagnostic value of urinary monocyte chemoattractant protein-1 in evaluating the activity of lupus nephritis: a meta-analysis. Lupus. 2020;29(6):599-606.
2. Zhang T, Duran V, Vanarsa K, Mohan C. Targeted urine proteomics in lupus nephritis - a meta-analysis. Expert Rev Proteomics. 2020;17(10):767-76.
3. JBI. Critical Appraisal Tools. Available online: https://jbi.global/critical-appraisal-tools (accessed on 4 July 2022)
