# Supplementary material for: Current Insights on Biomarkers in Lupus Nephritis: A Systematic Review of the Literature
Source: J Clin Med. 2022 Sep 28;11(19):5759. doi: 10.3390/jcm11195759 (PMC9570701; doi:10.3390/jcm11195759)
Supplement: Supplementary file 1 [file jcm-11-05759-s001.zip › jcm-1917751-supplementary-updated/Table S4.pdf]

Table S4. Risk of bias assessment of cohort studies*.

Author, year	Were the two groups similar and recruited from the same population?	Were the exposures measured similarly to assign people to both exposed and unexposed groups?	Was the exposure measured in a valid and reliable way?	Were confounding factors identified?	Were strategies to deal with confounding factors stated?	Were the groups/participants free of the outcome at the start of the study (or at the moment of exposure)?	Were the outcomes measured in a valid and reliable way?	Was the follow up time reported and sufficient to be long enough for outcomes to occur?	Was follow up complete, and if not, were the reasons to loss to follow up described and explored?	Were strategies to address incomplete follow up utilized?	Was appropriate statistical analysis used?
Aggarwal et al., 2017 [1]	Yes	Not applicable	Not applicable	Yes	Yes	No	Yes	Yes	Unclear	Unclear	Yes
Bona et al., 2020 [2]	Yes	Not applicable	Not applicable	No	No	No	Yes	Unclear	Unclear	Not applicable	Yes
Buyon et al., 2017 [3]	Yes	Not applicable	Not applicable	Yes	Yes	Yes	Yes	Yes	Unclear	Unclear	Yes
Carlsson et al., 2022 [4]	Yes	Not applicable	No	No	No	Not applicable	Yes	Yes	Unclear	Unclear	No
Chen et al., 2019 [5]	Yes	Yes	Yes	Yes	Yes	Yes	Yes	Yes	Unclear	Unclear	Yes
Cheng et al., 2015 [6]	Yes	Not applicable	Not applicable	Yes	Yes	Yes	Yes	Yes	Unclear	Unclear	Yes
Davies et al., [7]	Yes	Not applicable	Yes	Yes	Yes	Yes	Yes	Yes	Unclear	Unclear	Yes
Davies et al., [8]	Yes	Not applicable	Not applicable	Yes	Yes	Yes	Yes	Yes	Unclear	Unclear	Yes
Dedong et al., 2019 [9]	Yes	Not applicable	Not applicable	No	No	Yes	Yes	Unclear	Yes	Not applicable	Yes
Ding et al., 2021 [10]	Not applicable	Not applicable	Not applicable	Yes	Yes	Yes	Yes	Yes	No	No	Yes
Fasano et al., 2021 [11]	Not applicable	Not applicable	Not applicable	No	No	Yes	Yes	Yes	Yes	Not applicable	Yes
Fatemi et al., 2016 [12]	Yes	Not applicable	Not applicable	No	No	Yes	Yes	Yes	Yes	Not applicable	Yes
Fava et al., 2021[13]	Yes	Not applicable	Not applicable	Yes	Yes	Yes	Not applicable	Yes	Unclear	Not applicable	Yes
Garcia-Vives et al., 2020 [14]	Yes	Not applicable	Not applicable	No	No	Yes	Yes	Yes	Yes	Not applicable	Yes
Kim et al., 2020 [15]	Not applicable	Not applicable	Not applicable	Yes	Yes	Yes	Yes	Yes	Yes	Unclear	Yes

Kwon et al., 2020 [16]	Not applicable	Not applicable	Not applicable	No	No	Yes	Yes	Yes	Yes	Not applicable	Yes
Leatherwood et al., 2019 [17]	Not applicable	Not applicable	Not applicable	Yes	Yes	Yes	Yes	Yes	Yes	Yes	Yes
Li et al., 2019 [18]	Yes	Not applicable	Not applicable	Yes	Yes	Yes	Yes	Yes	Unclear	Unclear	Yes
Liu et al., 2020 [19]	Yes	Not applicable	Not applicable	Yes	Yes	Yes	Yes	Yes	Yes	Not applicable	Yes
Liu et al., 2021[20]	Yes	Not applicable	Not applicable	Yes	No	Yes	Yes	Yes	Yes	Not applicable	Yes
Martin et al., 2020 [21]	No	Not applicable	Yes	No	No	Yes	Yes	Yes	Yes	Not applicable	Yes
Mejia-Vilet et al., 2020 [22]	Not applicable	Not applicable	Not applicable	No	No	Yes	Yes	Yes	Yes	Not applicable	Yes
Mejia-Vilet et al., 2021[23]	No	Not applicable	Not applicable	Yes	Yes	Not applicable	Yes	Yes	Yes	Not applicable	Yes
Parodis et al., 2015 [24]	Not applicable	Not applicable	Yes	Yes	Yes	Yes	Yes	Yes	Yes	Not applicable	Yes
Parodis et al., 2017 [25]	Yes	Not applicable	Yes	Yes	Yes	Yes	Yes	Yes	Yes	Not applicable	Yes
Parodis et al., 2019 [26]	Yes	Not applicable	Yes	Yes	Yes	No	Yes	Yes	Yes	Yes	Yes
Parodis et al., 2020 [27]	Yes	Not applicable	No	Yes	Yes	Yes	Yes	Yes	Yes	Yes	Yes
Petri et al., 2021 [28]	Not applicable	Not applicable	Not applicable	Yes	Yes	Yes	Yes	Yes	Unclear	Unclear	Yes
Phatak et al., 2017 [29]	Yes	Yes	Yes	No	No	Yes	Yes	Yes	No	Unclear	No
Ren et al., 2018 [30]	Not applicable	Not applicable	Not applicable	Yes	Yes	Yes	Yes	Yes	Unclear	Unclear	Yes
Ruchakorn et al., 2019 [31]	Yes	Not applicable	Not applicable	Yes	Yes	No	Yes	Yes	Unclear	Unclear	Yes
Satirapoj et al., 2017 [32]	Not applicable	Yes	Yes	No	No	Yes	Yes	Yes	Yes	Not applicable	Yes
Torres-Salido et al., 2019 [33]	Not applicable	Not applicable	Yes	No	No	Yes	Yes	Yes	Unclear	Not applicable	Yes

Treamtrakanpon et al., 2012 [34]	Not applicable	Not applicable	Yes	No	No	Yes	Yes	Yes	Yes	Not applicable	Yes
Vincent et al., 2018 [35]	No	Not applicable	Not applicable	Yes	Yes	Yes	Yes	Yes	Unclear	Unclear	Yes
Wang et al., 2016 [36]	Yes	Yes	No	Yes	Yes	Yes	Yes	Yes	Yes	Not applicable	Yes
Wang et al., 2018 [37]	Not applicable	Not applicable	Not applicable	Yes	Yes	Yes	Yes	Yes	Yes	Not applicable	Yes
Wang et al., 2020 [38]	Unclear	Not applicable	Not applicable	Yes	Yes	Yes	Yes	Yes	Yes	Not applicable	Yes
Wolf et al., 2016 [39]	Not applicable	Not applicable	Yes	No	No	Yes	Yes	Yes	Unclear	Not applicable	Yes
Yap et al., 2016 [40]	Not applicable	Not applicable	Not applicable	No	No	No	Yes	Yes	Yes	Not applicable	Yes
Yu et al., 2021 [41]	Not applicable	Not applicable	Not applicable	No	No	Yes	Yes	Yes	Unclear	Not applicable	Yes

* Assessed by Joanna Briggs Institute (JBI) Critical Appraisal Checklist for Cohort Studies [42].

References

1. Aggarwal, A.; Gupta, R.; Negi, V.S.; Rajasekhar, L.; Misra, R.; Singh, P.; Chaturvedi, V.; Sinha, S. Urinary haptoglobin, alpha-1 anti-chymotrypsin and retinol binding protein identified by proteomics as potential biomarkers for lupus nephritis. *Clinical and experimental immunology* **2017**, *188*, 254-262.
2. Bona, N.; Pezzarini, E.; Balbi, B.; Daniele, S.M.; Rossi, M.F.; Monje, A.L.; Basiglio, C.L.; Pelusa, H.F.; Arriaga, S.M.M. Oxidative stress, inflammation and disease activity biomarkers in lupus nephropathy. *Lupus* **2020**, *29*, 311-323, doi:https://dx.doi.org/10.1177/0961203320904784.
3. Buyon, J.P.; Kim, M.Y.; Guerra, M.M.; Lu, S.; Reeves, E.; Petri, M.; Laskin, C.A.; Lockshin, M.D.; Sammaritano, L.R.; Branch, D.W.; et al. Kidney Outcomes and Risk Factors for Nephritis (Flare/De Novo) in a Multiethnic Cohort of Pregnant Patients with Lupus. *Clinical journal of the American Society of Nephrology : CJASN* **2017**, *12*, 940-946.
4. Carlsson, E.; Quist, A.; Davies, J.C.; Midgley, A.; Smith, E.M.D.; Bruce, I.N.; Beresford, M.W.; Hedrich, C.M.; Consortia, B.-B.a.M.M. Longitudinal analysis of urinary proteins in lupus nephritis - A pilot study. *Clinical immunology (Orlando, Fla.)* **2022**, *236*, 108948.
5. Chen, Y.M.; Hung, W.T.; Liao, Y.W.; Hsu, C.Y.; Hsieh, T.Y.; Chen, H.H.; Hsieh, C.W.; Lin, C.T.; Lai, K.L.; Tang, K.T.; et al. Combination immunosuppressant therapy and lupus nephritis outcome: a hospital-based study. *Lupus* **2019**, *28*, 658-666.
6. Cheng, F.-J.; Zhou, X.-J.; Zhao, Y.-F.; Zhao, M.-H.; Zhang, H. Human neutrophil peptide 1-3, a component of the neutrophil extracellular trap, as a potential biomarker of lupus nephritis. *International journal of rheumatic diseases* **2015**, *18*, 533-540.

7. Davies, J.C.; Midgley, A.; Carlsson, E.; Donohue, S.; Bruce, I.N.; Beresford, M.W.; Hedrich, C.M. Urine and serum S100A8/A9 and S100A12 associate with active lupus nephritis and may predict response to rituximab treatment. *RMD open* **2020**, *6*.
8. Davies, J.C.; Carlsson, E.; Midgley, A.; Smith, E.M.D.; Bruce, I.N.; Beresford, M.W.; Hedrich, C.M.; Consortia, B.-B.a.M.M. A panel of urinary proteins predicts active lupus nephritis and response to rituximab treatment. *Rheumatology (Oxford, England)* **2021**, *60*, 3747-3759.
9. Dedong, H.; Feiyan, Z.; Jie, S.; Xiaowei, L.; Shaoyang, W. Analysis of interleukin-17 and interleukin-23 for estimating disease activity and predicting the response to treatment in active lupus nephritis patients. *Immunology letters* **2019**, *210*, 33-39.
10. Ding, Y.; Yu, X.; Wu, L.; Tan, Y.; Qu, Z.; Yu, F. The Spectrum of C4d Deposition in Renal Biopsies of Lupus Nephritis Patients. *Frontiers in immunology* **2021**, *12*, 654652.
11. Fasano, S.; Pierro, L.; Borgia, A.; Coscia, M.A.; Formica, R.; Bucci, L.; Riccardi, A.; Ciccia, F. Biomarker panels may be superior over single molecules in prediction of renal flares in systemic lupus erythematosus: an exploratory study. *Rheumatology (Oxford, England)* **2020**, *59*, 3193-3200.
12. Fatemi, A.; Samadi, G.; Sayedbonakdar, Z.; Smiley, A. Anti-C1q antibody in patients with lupus nephritic flare: 18-month follow-up and a nested case-control study. *Modern rheumatology* **2016**, *26*, 233-239.
13. Fava, A.; Rao, D.A.; Mohan, C.; Zhang, T.; Rosenberg, A.; Fenaroli, P.; Belmont, H.M.; Izmirly, P.; Clancy, R.; Trujillo, J.M.; et al. Urine Proteomics and Renal Single-Cell Transcriptomics Implicate Interleukin-16 in Lupus Nephritis. *Arthritis Rheumatol* **2022**, *74*, 829-839, doi:10.1002/art.42023.
14. Garcia-Vives, E.; Sole, C.; Moline, T.; Vidal, M.; Agraz, I.; Ordi-Ros, J.; Cortes-Hernandez, J. The Urinary Exosomal miRNA Expression Profile is Predictive of Clinical Response in Lupus Nephritis. *International journal of molecular sciences* **2020**, *21*.
15. Kim, H.; Kim, T.; Kim, M.; Lee, H.Y.; Kim, Y.; Kang, M.S.; Kim, J. Activation of the alternative complement pathway predicts renal outcome in patients with lupus nephritis. *Lupus* **2020**, *29*, 862-871.
16. Kwon, O.C.; Lee, E.-J.; Oh, J.S.; Hong, S.; Lee, C.-K.; Yoo, B.; Park, M.-C.; Kim, Y.-G. Plasma immunoglobulin binding protein 1 as a predictor of development of lupus nephritis. *Lupus* **2020**, *29*, 547-553.
17. Leatherwood, C.; Speyer, C.B.; Feldman, C.H.; D'Silva, K.; Gómez-Puerta, J.A.; Hoover, P.J.; Waikar, S.S.; McMahon, G.M.; Rennke, H.G.; Costenbader, K.H. Clinical characteristics and renal prognosis associated with interstitial fibrosis and tubular atrophy (IFTA) and vascular injury in lupus nephritis biopsies. *Semin Arthritis Rheum* **2019**, *49*, 396-404, doi:10.1016/j.semarthrit.2019.06.002.
18. Li, Y.-J.; Wu, H.-H.; Liu, S.-H.; Tu, K.-H.; Lee, C.-C.; Hsu, H.-H.; Chang, M.-Y.; Yu, K.-H.; Chen, W.; Tian, Y.-C. Polyomavirus BK, BKV microRNA, and urinary neutrophil gelatinase-associated lipocalin can be used as potential biomarkers of lupus nephritis. *PloS one* **2019**, *14*, e0210633.
19. Liu, L.; Wang, R.; Ding, H.; Tian, L.; Gao, T.; Bao, C. The utility of urinary biomarker panel in predicting renal pathology and treatment response in Chinese lupus nephritis patients. *PloS one* **2020**, *15*, e0240942.
20. Liu, X.-R.; Qi, Y.-Y.; Zhao, Y.-F.; Cui, Y.; Wang, X.-Y.; Zhao, Z.-Z. Albumin-to-globulin ratio (AGR) as a potential marker of predicting lupus nephritis in Chinese patients with systemic lupus erythematosus. *Lupus* **2021**, *30*, 412-420.
21. Martin, M.; Trattner, R.; Nilsson, S.C.; Bjork, A.; Zickert, A.; Blom, A.M.; Gunnarsson, I. Plasma C4d Correlates With C4d Deposition in Kidneys and With Treatment Response in Lupus Nephritis Patients. *Frontiers in immunology* **2020**, *11*, 582737.
22. Mejia-Vilet, J.M.; Zhang, X.L.; Cruz, C.; Cano-Verduzco, M.L.; Shapiro, J.P.; Nagaraja, H.N.; Morales-Buenrostro, L.E.; Rovin, B.H. Urinary Soluble CD163: a Novel Noninvasive Biomarker of Activity for Lupus Nephritis. *Journal of the American Society of Nephrology : JASN* **2020**, *31*, 1335-1347.

23. Mejia-Vilet, J.M.; Shapiro, J.P.; Zhang, X.L.; Cruz, C.; Zimmerman, G.; Mendez-Perez, R.A.; Cano-Verduzco, M.L.; Parikh, S.V.; Nagaraja, H.N.; Morales-Buenrostro, L.E.; et al. Association Between Urinary Epidermal Growth Factor and Renal Prognosis in Lupus Nephritis. *Arthritis & rheumatology (Hoboken, N.J.)* **2021**, *73*, 244-254.
24. Parodis, I.; Zickert, A.; Sundelin, B.; Axelsson, M.; Gerhardsson, J.; Svenungsson, E.; Malmstrom, V.; Gunnarsson, I. Evaluation of B lymphocyte stimulator and a proliferation inducing ligand as candidate biomarkers in lupus nephritis based on clinical and histopathological outcome following induction therapy. *Lupus Sci Med* **2015**, *2*, e000061, doi:10.1136/lupus-2014-000061.
25. Parodis, I.; Ding, H.; Zickert, A.; Arnaud, L.; Larsson, A.; Svenungsson, E.; Mohan, C.; Gunnarsson, I. Serum soluble tumour necrosis factor receptor-2 (sTNFR2) as a biomarker of kidney tissue damage and long-term renal outcome in lupus nephritis. *Scandinavian Journal of Rheumatology* **2017**, *46*, 263-272, doi:https://dx.doi.org/10.1080/03009742.2016.1231339.
26. Parodis, I.; Ding, H.; Zickert, A.; Cosson, G.; Fathima, M.; Gronwall, C.; Mohan, C.; Gunnarsson, I. Serum Axl predicts histology-based response to induction therapy and long-term renal outcome in lupus nephritis. *PloS one* **2019**, *14*, e0212068.
27. Parodis, I.; Gokaraju, S.; Zickert, A.; Vanarsa, K.; Zhang, T.; Habazi, D.; Botto, J.; Serdoura Alves, C.; Giannopoulos, P.; Larsson, A.; et al. ALCAM and VCAM-1 as urine biomarkers of activity and long-term renal outcome in systemic lupus erythematosus. *Rheumatology (Oxford, England)* **2020**, *59*, 2237-2249.
28. Petri, M.; Barr, E.; Magder, L.S. Risk of Renal Failure Within 10 or 20 Years of Systemic Lupus Erythematosus Diagnosis. *The Journal of rheumatology* **2021**, *48*, 222-227.
29. Phatak, S.; Chaurasia, S.; Mishra, S.K.; Gupta, R.; Agrawal, V.; Aggarwal, A.; Misra, R. Urinary B cell activating factor (BAFF) and a proliferation-inducing ligand (APRIL): potential biomarkers of active lupus nephritis. *Clinical and experimental immunology* **2017**, *187*, 376-382.
30. Ren, Y.; Xie, J.; Lin, F.; Luo, W.; Zhang, Z.; Mao, P.; Zhong, R.; Liang, Y.; Yang, Z. Serum human epididymis protein 4 is a predictor for developing nephritis in patients with systemic lupus erythematosus: A prospective cohort study. *International immunopharmacology* **2018**, *60*, 189-193.
31. Ruchakorn, N.; Ngamjanyaporn, P.; Suangtamai, T.; Kafaksom, T.; Polpanumas, C.; Petpisit, V.; Pisitkun, T.; Pisitkun, P. Performance of cytokine models in predicting SLE activity. *Arthritis research & therapy* **2019**, *21*, 287.
32. Satirapoj, B.; Kitiyakara, C.; Leelahavanichkul, A.; Avihingsanon, Y.; Supasyndh, O. Urine neutrophil gelatinase-associated lipocalin to predict renal response after induction therapy in active lupus nephritis. *BMC nephrology* **2017**, *18*, 263.
33. Torres-Salido, M.T.; Sanchis, M.; Sole, C.; Moline, T.; Vidal, M.; Vidal, X.; Sola, A.; Hotter, G.; Ordi-Ros, J.; Cortes-Hernandez, J. Urinary Neuropilin-1: A Predictive Biomarker for Renal Outcome in Lupus Nephritis. *International journal of molecular sciences* **2019**, *20*.
34. Treamtrakanpon, W.; Tantivitayakul, P.; Benjachat, T.; Somparn, P.; Kittikowit, W.; Eiam-ong, S.; Leelahavanichkul, A.; Hirankarn, N.; Avihingsanon, Y. APRIL, a proliferation-inducing ligand, as a potential marker of lupus nephritis. *Arthritis research & therapy* **2012**, *14*, R252.
35. Vincent, F.B.; Kandane-Rathnayake, R.; Hoi, A.Y.; Slavin, L.; Godsell, J.D.; Kitching, A.R.; Harris, J.; Nelson, C.L.; Jenkins, A.J.; Chrysostomou, A.; et al. Urinary B-cell-activating factor of the tumour necrosis factor family (BAFF) in systemic lupus erythematosus. *Lupus* **2018**, *27*, 2029-2040.
36. Wang, Y.; Huang, X.; Cai, J.; Xie, L.; Wang, W.; Tang, S.; Yin, S.; Gao, X.; Zhang, J.; Zhao, J.; et al. Clinicopathologic Characteristics and Outcomes of Lupus Nephritis With Antineutrophil Cytoplasmic Antibody: A Retrospective Study. *Medicine* **2016**, *95*, e2580.
37. Wang, S.; Wu, M.; Chiriboga, L.; Zeck, B.; Belmont, H.M. Membrane attack complex (mac) deposition in lupus nephritis is associated with hypertension and poor clinical response to treatment. *Seminars in arthritis and rheumatism* **2018**, *48*, 256-262.

38. Wang, S.; Shang, J.; Xiao, J.; Zhao, Z. Clinicopathologic characteristics and outcomes of lupus nephritis with positive antineutrophil cytoplasmic antibody. *Renal failure* **2020**, *42*, 244-254.
39. Wolf, B.J.; Spainhour, J.C.; Arthur, J.M.; Janech, M.G.; Petri, M.; Oates, J.C. Development of Biomarker Models to Predict Outcomes in Lupus Nephritis. *Arthritis & rheumatology (Hoboken, N.J.)* **2016**, *68*, 1955-1963.
40. Yap, D.Y.H.; Yung, S.; Zhang, Q.; Tang, C.; Chan, T.M. Serum level of proximal renal tubular epithelial cell-binding immunoglobulin G in patients with lupus nephritis. *Lupus* **2016**, *25*, 46-53.
41. Yu, K.Y.C.; Yung, S.; Chau, M.K.M.; Tang, C.S.O.; Yap, D.Y.H.; Tang, A.H.N.; Ying, S.K.Y.; Lee, C.K.; Chan, T.M. Serum syndecan-1, hyaluronan and thrombomodulin levels in patients with lupus nephritis. *Rheumatology (Oxford, England)* **2021**, *60*, 737-750.
42. JBI. Critical Appraisal Tools. Available online: https://jbi.global/critical-appraisal-tools. (accessed on 4 July 2022).
