# Supplementary material for: Current Insights on Biomarkers in Lupus Nephritis: A Systematic Review of the Literature
Source: J Clin Med. 2022 Sep 28;11(19):5759. doi: 10.3390/jcm11195759 (PMC9570701; doi:10.3390/jcm11195759)
Supplement: Supplementary file 1 [file jcm-11-05759-s001.zip › jcm-1917751-supplementary-updated/Table S5.pdf]

Table S5. Ethnicity and/or nationality of the populations in the included studies.

Study	Year	N	Ethnicity
Aggarwal et al. [1]	2017	88	N/A
Alharazy et al. [2]	2013	100	Asian (Malaysian, Chinese, Indian)
Alharazy et al. [3]	2015	100	Asian (Malaysian, Chinese, Indian)
Alves et al. [4]	2021	41	N/A
Barbado et al. [5]	2012	48	N/A
Barnado et al. [6]	2019	1097	White/Caucasian; Black/African American; Asian; Hispanic/Latin American
Birmingham et al. [7]	2016	114	White/Caucasian; Black/African American
Bock et al. [8]	2015	52	N/A
Bona et al. [9]	2020	65	N/A
Bruschi et al. [10]	2021	1052	N/A
Burbano et al. [11]	2019	60	N/A
Buyon et al. [12]	2017	385	White/Caucasian; Black/African American; Asian; Hispanic/Latin American
Calich et al. [13]	2018	46	N/A
Carlsson et al. [14]	2022	25	White/Caucasian; Black/African American; Asian; Hispanic/Latin American
Chalmers et al. [15]	2022	788	White/Caucasian; Black/African American; Asian; Hispanic/Latin American
Chen et al. [16]	2019	148	N/A
Cheng et al. [17]	2015	80	N/A
Choe et al. [18]	2014	64	N/A
Choe et al. [19]	2016	70	N/A
Davies et al. [20]	2020	243	White/Caucasian; Black/African American; Asian; Hispanic/Latin American
Davies et al. [21]	2021	197	White/Caucasian; Black/African American; Asian; Hispanic/Latin American
Dedong et al. [22]	2019	80	N/A
Ding et al. [23]	2016	85	White/Caucasian; Black/African American; Asian; Hispanic/Latin American
Ding et al. [24]	2020	256	Chinese
Ding et al. [25]	2021	325	N/A
Dolff et al. [26]	2013	46	N/A
Dong et al. [27]	2018	70	Chinese
Elsaid et al. [28]	2021	92	N/A
Endo et al. [29]	2016	74	N/A
Enghard et al. [30]	2014	147	N/A
Fasano et al. [31]	2020	61	N/A
Fatemi et al. [32]	2016	69	N/A
Fava et al. [33]	2022	131	White/Caucasian; Black/African American; Asian; Hispanic/Latin American
Garcia-Vives et al. [34]	2020	14	White/Caucasian; Hispanic/Latin American
Go et al. [35]	2018	121	N/A
Gomez-Puerta et al. [36]	2018	120	Mestizo (European and Amerindian ancestry); Afro-Latin American; White/Caucasian; Colombian Amerindian
Hafez et al. [37]	2021	60	Egyptian
Hardt et al. [38]	2018	623	White/Caucasian
Howe et al. [39]	2012	121	Asian
Huang et al. [40]	2019	144	N/A

Hutcheson et al. [41]	2015	38	White/Caucasian; Black/African American; Asian; Hispanic/Latin American
Ichinose et al. [42]	2018	73	N/A
Ishizaki et al. [43]	2015	182	N/A
Jakiela et al. [44]	2018	33	N/A
Khoshmirsafa et al. [45]	2019	55	N/A
Kianmehr et al. [46]	2021	95	N/A
Kim et al. [47]	2020	115	N/A
Koo et al. [48]	2016	193	Korean
Kwon et al. [49]	2020	43	N/A
Leatherwood et al. [50]	2019	202	Black/African American; Non-Black/African American
Li et al. [51]	2019	90	N/A
Liu et al. [52]	2020	154	Chinese
Liu et al. [53]	2021	194	N/A
Martin et al. [54]	2017	93	White/Caucasian; Black/African American; Asian; Hispanic/Latin American
Mejia-Vilet et al. [55]	2020	511	White/Caucasian; Black/African American; Mexican
Mejia-Vilet et al. [56]	2021	367	White/Caucasian; Black/African American; Mexican
Menke et al. [57]	2015	264	White/Caucasian; Black/African American; Asian; Hispanic/Latin American
Mirioglu et al. [58]	2020	51	N/A
Mok et al. [59]	2012	290	N/A
Mok et al. [60]	2016	94	N/A
Mok et al. [61]	2018	227	Chinese
Nakhjavani et al. [62]	2019	26	N/A
Nordin et al. [63]	2019	120	Malaysian
Pang et al. [64]	2018	470	N/A
Parodis et al. [65]	2015	64	White/Caucasian (mainly); Black/African American; Asian; Hispanic/Latin American
Parodis et al. [66]	2017	64	White/Caucasian (mainly); Black/African American; Asian; Hispanic/Latin American
Parodis et al. [67]	2019	64	White/Caucasian (mainly); Black/African American; Asian
Parodis et al. [68]	2020	111	White/Caucasian
Petri et al. [69]	2021	2528	White/Caucasian; Black/African American; Asian; Hispanic/Latin American
Phatak et al. [70]	2017	46	N/A
Qin et al. [71]	2019	133	White/Caucasian; Black/African American; Asian; Hispanic/Latin American
Ren et al. [72]	2018	74	N/A
Reyes-Martinez et al. [73]	2018	44	Mexican
Rosa et al. [74]	2012	75	Brazilian
Ruchakorn et al. [75]	2019	124	N/A
Salem et al. [76]	2018	44	N/A
Satirapoj et al. [77]	2017	68	N/A
Selvaraja et al. [78]	2019	64	Malaysian
Singh et al. [79]	2012	74	White/Caucasian; Black/African American; Hispanic/Latin American
Sjowall et al. [80]	2018	261	White/Caucasian

Smith et al. [81]	2019	189	White/Caucasian; Black/African American; Asian; Hispanic/Latin American
Stanley et al. [82]	2019	132	White/Caucasian; Black/African American; Asian (Chinese); Hispanic/Latin American
Stanley et al. [83]	2020	301	White/Caucasian; Black/African American; Asian (Chinese)
Taha et al. [84]	2017	42	N/A
Tang et al. [85]	2022	62	White/Caucasian; Black/African American; Asian; Hispanic/Latin American
Torres-Salido et al. [86]	2019	95	White/Caucasian; Hispanic/Latin American
Treamtrakanpon et al. [87]	2012	47	N/A
Urrego et al. [88]	2020	120	Mestizo (European and Amerindian ancestry); Afro-Latin American
Urrego-Callejas et al. [89]	2021	120	Mestizo (European and Amerindian ancestry); Afro-Latin American
Vanarsa et al. [90]	2020	82	White/Caucasian; Black/African American; Hispanic/Latin American
Vincent et al. [91]	2018	85	White/Caucasian; Asian
Wang et al. [92]	2016	154	Chinese
Wang et al. [93]	2018	192	Chinese
Wang et al. [94]	2018	30	White/Caucasian; Black/African American; Asian; Hispanic/Latin American
Wang et al. [95]	2020	283	Chinese
Wantanasiri et al. [96]	2015	42	N/A
Wolf et al. [97]	2016	140	White/Caucasian; Black/African American
Wu et al. [98]	2013	100	White/Caucasian; Black/African American; Hispanic/Latin American
Wu et al. [99]	2016	154	N/A
Wu et al. [100]	2016	85	White/Caucasian; Black/African American; Asian; Hispanic/Latin American
Wu et al. [101]	2016	86	White/Caucasian; Black/African American; Asian; Hispanic/Latin American
Xia et al. [102]	2020	521	White/Caucasian; Black/African American; Asian; Hispanic/Latin American; Mestizo (European and Amerindian ancestry)
Yang et al. [103]	2016	209	Chinese
Yap et al. [104]	2016	23	Chinese
Yu et al. [105]	2021	31	Chinese
Zhang et al. [106]	2020	228	White/Caucasian; Black/African American; Asian
Zhang et al. [107]	2020	N/A	N/A

The total number of participants in each study is indicated by N.

N/A: not applicable

References

1. Aggarwal, A.; Gupta, R.; Negi, V.S.; Rajasekhar, L.; Misra, R.; Singh, P.; Chaturvedi, V.; Sinha, S. Urinary haptoglobin, alpha-1 anti-chymotrypsin and retinol binding protein identified by proteomics as potential biomarkers for lupus nephritis. *Clinical and experimental immunology* **2017**, *188*, 254-262.
2. Alharazy, S.M.; Kong, N.C.T.; Mohd, M.; Shah, S.A.; Abdul Gafor, A.H.; Ba'in, A. The role of urinary neutrophil gelatinase-associated lipocalin in lupus nephritis. *Clinica chimica acta; international journal of clinical chemistry* **2013**, *425*, 163-168.
3. Alharazy, S.; Kong, N.C.; Mohd, M.; Shah, S.A.; Ba'in, A.; Abdul Gafor, A.H. Urine Monocyte Chemoattractant Protein-1 and Lupus Nephritis Disease Activity: Preliminary Report of a Prospective Longitudinal Study. *Autoimmune Dis* **2015**, *2015*, 962046, doi:10.1155/2015/962046.
4. Alves, I.; Santos-Pereira, B.; Dalebout, H.; Santos, S.; Vicente, M.M.; Campar, A.; Thepaut, M.; Fieschi, F.; Strahl, S.; Boyaval, F.; et al. Protein Mannosylation as a Diagnostic and Prognostic Biomarker of Lupus Nephritis: An Unusual Glycan Neoepitope in Systemic Lupus Erythematosus. *Arthritis & rheumatology (Hoboken, N.J.)* **2021**, *73*, 2069-2077.
5. Barbado, J.; Martin, D.; Vega, L.; Almansa, R.; Goncalves, L.; Nocito, M.; Jimeno, A.; Ortiz de Lejarazu, R.; Bermejo-Martin, J.F. MCP-1 in urine as biomarker of disease activity in Systemic Lupus Erythematosus. *Cytokine* **2012**, *60*, 583-586.
6. Barnado, A.; Carroll, R.J.; Casey, C.; Wheless, L.; Denny, J.C.; Crofford, L.J. Phenome-wide association study identifies dsDNA as a driver of major organ involvement in systemic lupus erythematosus. *Lupus* **2019**, *28*, 66-76.
7. Birmingham, D.J.; Bitter, J.E.; Ndukwe, E.G.; Dials, S.; Gullo, T.R.; Conroy, S.; Nagaraja, H.N.; Rovin, B.H.; Hebert, L.A. Relationship of Circulating Anti-C3b and Anti-C1q IgG to Lupus Nephritis and Its Flare. *Clinical journal of the American Society of Nephrology : CJASN* **2016**, *11*, 47-53.
8. Bock, M.; Heijnen, I.; Trendelenburg, M. Anti-C1q antibodies as a follow-up marker in SLE patients. *PloS one* **2015**, *10*, e0123572.
9. Bona, N.; Pezzarini, E.; Balbi, B.; Daniele, S.M.; Rossi, M.F.; Monje, A.L.; Basiglio, C.L.; Pelusa, H.F.; Arriaga, S.M.M. Oxidative stress, inflammation and disease activity biomarkers in lupus nephropathy. *Lupus* **2020**, *29*, 311-323.
10. Bruschi, M.; Moroni, G.; Sinico, R.A.; Franceschini, F.; Fredi, M.; Vaglio, A.; Cavagna, L.; Petretto, A.; Pratesi, F.; Migliorini, P.; et al. Serum IgG2 antibody multicomposition in systemic lupus erythematosus and lupus nephritis (Part 1): cross-sectional analysis. *Rheumatology (Oxford, England)* **2021**, *60*, 3176-3188.
11. Burbano, C.; Gomez-Puerta, J.A.; Munoz-Vahos, C.; Vanegas-Garcia, A.; Rojas, M.; Vasquez, G.; Castano, D. HMGB1+ microparticles present in urine are hallmarks of nephritis in patients with systemic lupus erythematosus. *European journal of immunology* **2019**, *49*, 323-335.
12. Buyon, J.P.; Kim, M.Y.; Guerra, M.M.; Lu, S.; Reeves, E.; Petri, M.; Laskin, C.A.; Lockshin, M.D.; Sammaritano, L.R.; Branch, D.W.; et al. Kidney Outcomes and Risk Factors for Nephritis (Flare/De Novo) in a Multiethnic Cohort of Pregnant Patients with Lupus. *Clinical journal of the American Society of Nephrology : CJASN* **2017**, *12*, 940-946.
13. Calich, A.L.; Borba, E.F.; Ugolini-Lopes, M.R.; da Rocha, L.F.; Bonfa, E.; Fuller, R. Serum uric acid levels are associated with lupus nephritis in patients with normal renal function. *Clinical rheumatology* **2018**, *37*, 1223-1228.
14. Carlsson, E.; Quist, A.; Davies, J.C.; Midgley, A.; Smith, E.M.D.; Bruce, I.N.; Beresford, M.W.; Hedrich, C.M.; Consortia, B.-B.a.M.M. Longitudinal analysis of urinary proteins in lupus nephritis - A pilot study. *Clinical immunology (Orlando, Fla.)* **2022**, *236*, 108948.
15. Chalmers, S.A.; Ayilam Ramachandran, R.; Garcia, S.J.; Der, E.; Herlitz, L.; Ampudia, J.; Chu, D.; Jordan, N.; Zhang, T.; Parodis, I.; et al. The CD6/ALCAM pathway promotes lupus nephritis via T cell-mediated responses. *J Clin Invest* **2022**, *132*, doi:10.1172/jci147334.
16. Chen, Y.M.; Hung, W.T.; Liao, Y.W.; Hsu, C.Y.; Hsieh, T.Y.; Chen, H.H.; Hsieh, C.W.; Lin, C.T.; Lai, K.L.; Tang, K.T.; et al. Combination immunosuppressant therapy and lupus nephritis outcome: a hospital-based study. *Lupus* **2019**, *28*, 658-666.
17. Cheng, F.-J.; Zhou, X.-J.; Zhao, Y.-F.; Zhao, M.-H.; Zhang, H. Human neutrophil peptide 1-3, a component of the neutrophil extracellular trap, as a potential biomarker of lupus nephritis. *International journal of rheumatic diseases* **2015**, *18*, 533-540.
18. Choe, J.Y.; Park, S.H.; Kim, S.K. Urine beta2-microglobulin is associated with clinical disease activity and renal involvement in female patients with systemic lupus erythematosus. *Lupus* **2014**, *23*, 1486-1493.
19. Choe, J.-Y.; Kim, S.-K. Serum TWEAK as a biomarker for disease activity of systemic lupus erythematosus. *Inflammation research : official journal of the European Histamine Research Society ... [et al.]* **2016**, *65*, 479-488.
20. Davies, J.C.; Midgley, A.; Carlsson, E.; Donohue, S.; Bruce, I.N.; Beresford, M.W.; Hedrich, C.M. Urine and serum S100A8/A9 and S100A12 associate with active lupus nephritis and may predict response to rituximab treatment. *RMD open* **2020**, *6*.
21. Davies, J.C.; Carlsson, E.; Midgley, A.; Smith, E.M.D.; Bruce, I.N.; Beresford, M.W.; Hedrich, C.M.; Consortia, B.-B.a.M.M. A panel of urinary proteins predicts active lupus nephritis and response to rituximab treatment. *Rheumatology (Oxford, England)* **2021**, *60*, 3747-3759.

22. Dedong, H.; Feiyan, Z.; Jie, S.; Xiaowei, L.; Shaoyang, W. Analysis of interleukin-17 and interleukin-23 for estimating disease activity and predicting the response to treatment in active lupus nephritis patients. *Immunology letters* **2019**, *210*, 33-39.
23. Ding, H.; Kharboutli, M.; Saxena, R.; Wu, T. Insulin-like growth factor binding protein-2 as a novel biomarker for disease activity and renal pathology changes in lupus nephritis. *Clinical and experimental immunology* **2016**, *184*, 11-18.
24. Ding, H.; Lin, C.; Cai, J.; Guo, Q.; Dai, M.; Mohan, C.; Shen, N. Urinary activated leukocyte cell adhesion molecule as a novel biomarker of lupus nephritis histology. *Arthritis Res Ther* **2020**, *22*, 122, doi:10.1186/s13075-020-02209-9.
25. Ding, Y.; Yu, X.; Wu, L.; Tan, Y.; Qu, Z.; Yu, F. The Spectrum of C4d Deposition in Renal Biopsies of Lupus Nephritis Patients. *Frontiers in immunology* **2021**, *12*, 654652.
26. Dolff, S.; Abdulahad, W.H.; Arends, S.; van Dijk, M.C.R.F.; Limburg, P.C.; Kallenberg, C.G.M.; Bijl, M. Urinary CD8+ T-cell counts discriminate between active and inactive lupus nephritis. *Arthritis research & therapy* **2013**, *15*, R36.
27. Dong, X.W.; Zheng, Z.H.; Ding, J.; Luo, X.; Li, Z.Q.; Li, Y.; Rong, M.Y.; Fu, Y.L.; Shi, J.H.; Yu, L.C.; et al. Combined detection of uMCP-1 and uTWEAK for rapid discrimination of severe lupus nephritis. *Lupus* **2018**, *27*, 971-981.
28. Elsaid, D.S.; Abdel Noor, R.A.; Shalaby, K.A.; Haroun, R.A.-H. Urinary Tumor Necrosis Factor-Like Weak Inducer of Apoptosis (uTWEAK) and Urinary Monocyte Chemo-attractant Protein-1 (uMCP-1): Promising Biomarkers of Lupus Nephritis Activity? *Saudi journal of kidney diseases and transplantation : an official publication of the Saudi Center for Organ Transplantation, Saudi Arabia* **2021**, *32*, 19-29.
29. Endo, N.; Tsuboi, N.; Furuhashi, K.; Shi, Y.; Du, Q.; Abe, T.; Hori, M.; Imaizumi, T.; Kim, H.; Katsuno, T.; et al. Urinary soluble CD163 level reflects glomerular inflammation in human lupus nephritis. *Nephrology, dialysis, transplantation : official publication of the European Dialysis and Transplant Association - European Renal Association* **2016**, *31*, 2023-2033.
30. Enghard, P.; Rieder, C.; Kopetschke, K.; Klocke, J.R.; Undeutsch, R.; Biesen, R.; Dragun, D.; Gollasch, M.; Schneider, U.; Aupperle, K.; et al. Urinary CD4 T cells identify SLE patients with proliferative lupus nephritis and can be used to monitor treatment response. *Annals of the rheumatic diseases* **2014**, *73*, 277-283.
31. Fasano, S.; Pierro, L.; Borgia, A.; Coscia, M.A.; Formica, R.; Bucci, L.; Riccardi, A.; Ciccia, F. Biomarker panels may be superior over single molecules in prediction of renal flares in systemic lupus erythematosus: an exploratory study. *Rheumatology (Oxford, England)* **2020**, *59*, 3193-3200.
32. Fatemi, A.; Samadi, G.; Sayedbonakdar, Z.; Smiley, A. Anti-C1q antibody in patients with lupus nephritic flare: 18-month follow-up and a nested case-control study. *Modern rheumatology* **2016**, *26*, 233-239.
33. Fava, A.; Rao, D.A.; Mohan, C.; Zhang, T.; Rosenberg, A.; Fenaroli, P.; Belmont, H.M.; Izmirly, P.; Clancy, R.; Trujillo, J.M.; et al. Urine Proteomics and Renal Single-Cell Transcriptomics Implicate Interleukin-16 in Lupus Nephritis. *Arthritis Rheumatol* **2022**, *74*, 829-839, doi:10.1002/art.42023.
34. Garcia-Vives, E.; Sole, C.; Moline, T.; Vidal, M.; Agraz, I.; Ordi-Ros, J.; Cortes-Hernandez, J. The Urinary Exosomal miRNA Expression Profile is Predictive of Clinical Response in Lupus Nephritis. *International journal of molecular sciences* **2020**, *21*.
35. Go, D.J.; Lee, J.Y.; Kang, M.J.; Lee, E.Y.; Lee, E.B.; Yi, E.C.; Song, Y.W. Urinary vitamin D-binding protein, a novel biomarker for lupus nephritis, predicts the development of proteinuric flare. *Lupus* **2018**, *27*, 1600-1615.
36. Gomez-Puerta, J.A.; Ortiz-Reyes, B.; Urrego, T.; Vanegas-Garcia, A.L.; Munoz, C.H.; Gonzalez, L.A.; Cervera, R.; Vasquez, G. Urinary neutrophil gelatinase-associated lipocalin and monocyte chemoattractant protein 1 as biomarkers for lupus nephritis in Colombian SLE patients. *Lupus* **2018**, *27*, 637-646.
37. Hafez, E.A.; Hassan, S.A.E.-M.; Teama, M.A.M.; Badr, F.M. Serum uric acid as a predictor for nephritis in Egyptian patients with systemic lupus erythematosus. *Lupus* **2021**, *30*, 378-384.
38. Hardt, U.; Larsson, A.; Gunnarsson, I.; Clancy, R.M.; Petri, M.; Buyon, J.P.; Silverman, G.J.; Svenungsson, E.; Gronwall, C. Autoimmune reactivity to malondialdehyde adducts in systemic lupus erythematosus is associated with disease activity and nephritis. *Arthritis research & therapy* **2018**, *20*, 36.
39. Howe, H.S.; Kong, K.O.; Thong, B.Y.H.; Law, W.G.; Chia, F.L.A.; Lian, T.Y.; Lau, T.C.; Chng, H.H.; Leung, B.P.L. Urine sVCAM-1 and sICAM-1 levels are elevated in lupus nephritis. *International journal of rheumatic diseases* **2012**, *15*, 13-16.
40. Huang, Y.; Chen, L.; Chen, K.; Huang, F.; Feng, Y.; Xu, Z.; Wang, W. Anti-alpha-enolase antibody combined with beta2 microglobulin evaluated the incidence of nephritis in systemic lupus erythematosus patients. *Lupus* **2019**, *28*, 365-370.
41. Hutcheson, J.; Ye, Y.; Han, J.; Arriens, C.; Saxena, R.; Li, Q.Z.; Mohan, C.; Wu, T. Resistin as a potential marker of renal disease in lupus nephritis. *Clinical and experimental immunology* **2015**, *179*, 435-443.
42. Ichinose, K.; Kitamura, M.; Sato, S.; Fujikawa, K.; Horai, Y.; Matsuoka, N.; Tsuboi, M.; Nonaka, F.; Shimizu, T.; Fukui, S.; et al. Podocyte foot process width is a prediction marker for complete renal response at 6 and 12months after induction therapy in lupus nephritis. *Clinical immunology (Orlando, Fla.)* **2018**, *197*, 161-168.

43. Ishizaki, J.; Saito, K.; Nawata, M.; Mizuno, Y.; Tokunaga, M.; Sawamukai, N.; Tamura, M.; Hirata, S.; Yamaoka, K.; Hasegawa, H.; et al. Low complements and high titre of anti-Sm antibody as predictors of histopathologically proven silent lupus nephritis without abnormal urinalysis in patients with systemic lupus erythematosus. *Rheumatology (Oxford, England)* **2015**, *54*, 405-412.
44. Jakiela, B.; Kosalka, J.; Plutecka, H.; Wegrzyn, A.S.; Bazan-Socha, S.; Sanak, M.; Musial, J. Urinary cytokines and mRNA expression as biomarkers of disease activity in lupus nephritis. *Lupus* **2018**, *27*, 1259-1270.
45. Khoshmirsafa, M.; Kianmehr, N.; Falak, R.; Mowla, S.J.; Seif, F.; Mirzaei, B.; Valizadeh, M.; Shekarabi, M. Elevated expression of miR-21 and miR-155 in peripheral blood mononuclear cells as potential biomarkers for lupus nephritis. *International journal of rheumatic diseases* **2019**, *22*, 458-467.
46. Kianmehr, N.; Khoshmirsafa, M.; Shekarabi, M.; Falak, R.; Haghighi, A.; Masoodian, M.; Seif, F.; Omidi, F.; Shirani, F.; Dadfar, N. High frequency of concurrent anti-C1q and anti-dsDNA but not anti-C3b antibodies in patients with Lupus Nephritis. *Journal of immunoassay & immunochemistry* **2021**, *42*, 406-423.
47. Kim, H.; Kim, T.; Kim, M.; Lee, H.Y.; Kim, Y.; Kang, M.S.; Kim, J. Activation of the alternative complement pathway predicts renal outcome in patients with lupus nephritis. *Lupus* **2020**, *29*, 862-871.
48. Koo, H.S.; Kim, S.; Chin, H.J. Remission of proteinuria indicates good prognosis in patients with diffuse proliferative lupus nephritis. *Lupus* **2016**, *25*, 3-11.
49. Kwon, O.C.; Lee, E.-J.; Oh, J.S.; Hong, S.; Lee, C.-K.; Yoo, B.; Park, M.-C.; Kim, Y.-G. Plasma immunoglobulin binding protein 1 as a predictor of development of lupus nephritis. *Lupus* **2020**, *29*, 547-553.
50. Leatherwood, C.; Speyer, C.B.; Feldman, C.H.; D'Silva, K.; Gómez-Puerta, J.A.; Hoover, P.J.; Waikar, S.S.; McMahon, G.M.; Rennke, H.G.; Costenbader, K.H. Clinical characteristics and renal prognosis associated with interstitial fibrosis and tubular atrophy (IFTA) and vascular injury in lupus nephritis biopsies. *Semin Arthritis Rheum* **2019**, *49*, 396-404, doi:10.1016/j.semarthrit.2019.06.002.
51. Li, Y.-J.; Wu, H.-H.; Liu, S.-H.; Tu, K.-H.; Lee, C.-C.; Hsu, H.-H.; Chang, M.-Y.; Yu, K.-H.; Chen, W.; Tian, Y.-C. Polyomavirus BK, BKV microRNA, and urinary neutrophil gelatinase-associated lipocalin can be used as potential biomarkers of lupus nephritis. *PloS one* **2019**, *14*, e0210633.
52. Liu, L.; Wang, R.; Ding, H.; Tian, L.; Gao, T.; Bao, C. The utility of urinary biomarker panel in predicting renal pathology and treatment response in Chinese lupus nephritis patients. *PloS one* **2020**, *15*, e0240942.
53. Liu, X.-R.; Qi, Y.-Y.; Zhao, Y.-F.; Cui, Y.; Wang, X.-Y.; Zhao, Z.-Z. Albumin-to-globulin ratio (AGR) as a potential marker of predicting lupus nephritis in Chinese patients with systemic lupus erythematosus. *Lupus* **2021**, *30*, 412-420.
54. Martin, M.; Smolag, K.I.; Bjork, A.; Gullstrand, B.; Okroj, M.; Leffler, J.; Jonsen, A.; Bengtsson, A.A.; Blom, A.M. Plasma C4d as marker for lupus nephritis in systemic lupus erythematosus. *Arthritis research & therapy* **2017**, *19*, 266.
55. Mejia-Vilet, J.M.; Zhang, X.L.; Cruz, C.; Cano-Verduzco, M.L.; Shapiro, J.P.; Nagaraja, H.N.; Morales-Buenrostro, L.E.; Rovin, B.H. Urinary Soluble CD163: a Novel Noninvasive Biomarker of Activity for Lupus Nephritis. *Journal of the American Society of Nephrology : JASN* **2020**, *31*, 1335-1347.
56. Mejia-Vilet, J.M.; Shapiro, J.P.; Zhang, X.L.; Cruz, C.; Zimmerman, G.; Mendez-Perez, R.A.; Cano-Verduzco, M.L.; Parikh, S.V.; Nagaraja, H.N.; Morales-Buenrostro, L.E.; et al. Association Between Urinary Epidermal Growth Factor and Renal Prognosis in Lupus Nephritis. *Arthritis & rheumatology (Hoboken, N.J.)* **2021**, *73*, 244-254.
57. Menke, J.; Amann, K.; Cavagna, L.; Blettner, M.; Weinmann, A.; Schwarting, A.; Kelley, V.R. Colony-stimulating factor-1: a potential biomarker for lupus nephritis. *Journal of the American Society of Nephrology : JASN* **2015**, *26*, 379-389.
58. Mirioglu, S.; Cinar, S.; Yazici, H.; Ozluk, Y.; Kilicaslan, I.; Gul, A.; Ocal, L.; Inanc, M.; Artim-Esen, B. Serum and urine TNF-like weak inducer of apoptosis, monocyte chemoattractant protein-1 and neutrophil gelatinase-associated lipocalin as biomarkers of disease activity in patients with systemic lupus erythematosus. *Lupus* **2020**, *29*, 379-388.
59. Mok, C.C.; Birmingham, D.J.; Ho, L.Y.; Hebert, L.A.; Song, H.; Rovin, B.H. Vitamin D deficiency as marker for disease activity and damage in systemic lupus erythematosus: a comparison with anti-dsDNA and anti-C1q. *Lupus* **2012**, *21*, 36-42.
60. Mok, C.C.; Ding, H.H.; Kharboutli, M.; Mohan, C. Axl, Ferritin, Insulin-Like Growth Factor Binding Protein 2, and Tumor Necrosis Factor Receptor Type II as Biomarkers in Systemic Lupus Erythematosus. *Arthritis care & research* **2016**, *68*, 1303-1309.
61. Mok, C.C.; Soliman, S.; Ho, L.Y.; Mohamed, F.A.; Mohamed, F.I.; Mohan, C. Urinary angiostatin, CXCL4 and VCAM-1 as biomarkers of lupus nephritis. *Arthritis research & therapy* **2018**, *20*, 6.
62. Nakhjavani, M.; Etemadi, J.; Pourlak, T.; Mirhosaini, Z.; Zununi Vahed, S.; Abediazar, S. Plasma levels of miR-21, miR-150, miR-423 in patients with lupus nephritis. *Iranian journal of kidney diseases* **2019**, *13*, 198-206.
63. Nordin, F.; Shaharir, S.S.; Abdul Wahab, A.; Mustafar, R.; Abdul Gafor, A.H.; Mohamed Said, M.S.; Rajalingham, S.; Shah, S.A. Serum and urine interleukin-17A levels as biomarkers of disease activity in systemic lupus erythematosus. *International journal of rheumatic diseases* **2019**, *22*, 1419-1426.

64. Pang, Y.; Tan, Y.; Li, Y.; Zhang, J.; Guo, Y.; Guo, Z.; Zhang, C.; Yu, F.; Zhao, M.-H. Serum A08 C1q antibodies are associated with disease activity and prognosis in Chinese patients with lupus nephritis. *Kidney international* **2016**, *90*, 1357-1367.
65. Parodis, I.; Zickert, A.; Sundelin, B.; Axelsson, M.; Gerhardsson, J.; Svenungsson, E.; Malmström, V.; Gunnarsson, I. Evaluation of B lymphocyte stimulator and a proliferation inducing ligand as candidate biomarkers in lupus nephritis based on clinical and histopathological outcome following induction therapy. *Lupus Sci Med* **2015**, *2*, e000061, doi:10.1136/lupus-2014-000061.
66. Parodis, I.; Ding, H.; Zickert, A.; Arnaud, L.; Larsson, A.; Svenungsson, E.; Mohan, C.; Gunnarsson, I. Serum soluble tumour necrosis factor receptor-2 (sTNFR2) as a biomarker of kidney tissue damage and long-term renal outcome in lupus nephritis. *Scandinavian journal of rheumatology* **2017**, *46*, 263-272.
67. Parodis, I.; Ding, H.; Zickert, A.; Cosson, G.; Fathima, M.; Gronwall, C.; Mohan, C.; Gunnarsson, I. Serum Axl predicts histology-based response to induction therapy and long-term renal outcome in lupus nephritis. *PloS one* **2019**, *14*, e0212068.
68. Parodis, I.; Gokaraju, S.; Zickert, A.; Vanarsa, K.; Zhang, T.; Habazi, D.; Botto, J.; Serdoura Alves, C.; Giannopoulos, P.; Larsson, A.; et al. ALCAM and VCAM-1 as urine biomarkers of activity and long-term renal outcome in systemic lupus erythematosus. *Rheumatology (Oxford, England)* **2020**, *59*, 2237-2249.
69. Petri, M.; Barr, E.; Magder, L.S. Risk of Renal Failure Within 10 or 20 Years of Systemic Lupus Erythematosus Diagnosis. *The Journal of rheumatology* **2021**, *48*, 222-227.
70. Phatak, S.; Chaurasia, S.; Mishra, S.K.; Gupta, R.; Agrawal, V.; Aggarwal, A.; Misra, R. Urinary B cell activating factor (BAFF) and a proliferation-inducing ligand (APRIL): potential biomarkers of active lupus nephritis. *Clinical and experimental immunology* **2017**, *187*, 376-382.
71. Qin, L.; Stanley, S.; Ding, H.; Zhang, T.; Truong, V.T.T.; Celhar, T.; Fairhurst, A.-M.; Pedroza, C.; Petri, M.; Saxena, R.; et al. Urinary pro-thrombotic, anti-thrombotic, and fibrinolytic molecules as biomarkers of lupus nephritis. *Arthritis research & therapy* **2019**, *21*, 176.
72. Ren, Y.; Xie, J.; Lin, F.; Luo, W.; Zhang, Z.; Mao, P.; Zhong, R.; Liang, Y.; Yang, Z. Serum human epididymis protein 4 is a predictor for developing nephritis in patients with systemic lupus erythematosus: A prospective cohort study. *International immunopharmacology* **2018**, *60*, 189-193.
73. Reyes-Martinez, F.; Perez-Navarro, M.; Rodriguez-Matias, A.; Soto-Abraham, V.; Gutierrez-Reyes, G.; Medina-Avila, Z.; Valdez-Ortiz, R. Assessment of urinary TWEAK levels in Mexican patients with untreated lupus nephritis: An exploratory study. *Nefrologia* **2018**, *38*, 152-160.
74. Rosa, R.F.; Takei, K.; Araujo, N.C.; Loduca, S.M.A.; Szajubok, J.C.M.; Chahade, W.H. Monocyte chemoattractant-1 as a urinary biomarker for the diagnosis of activity of lupus nephritis in Brazilian patients. *The Journal of rheumatology* **2012**, *39*, 1948-1954.
75. Ruchakorn, N.; Ngamjanyaporn, P.; Suangtamai, T.; Kafaksom, T.; Polpanumas, C.; Petpisit, V.; Pisitkun, T.; Pisitkun, P. Performance of cytokine models in predicting SLE activity. *Arthritis research & therapy* **2019**, *21*, 287.
76. Salem, M.N.; Taha, H.A.; Abd El-Fattah El-Feqi, M.; Eesa, N.N.; Mohamed, R.A. Urinary TNF-like weak inducer of apoptosis (TWEAK) as a biomarker of lupus nephritis. *TNF-ahnlicher schwacher Induktor von Apoptose (TWEAK) im Urin als Biomarker einer Lupusnephritis.* **2018**, *77*, 71-77.
77. Satirapoj, B.; Kitiyakara, C.; Leelahavanichkul, A.; Avihingsanon, Y.; Supasyndh, O. Urine neutrophil gelatinase-associated lipocalin to predict renal response after induction therapy in active lupus nephritis. *BMC nephrology* **2017**, *18*, 263.
78. Selvaraja, M.; Abdullah, M.; Arip, M.; Chin, V.K.; Shah, A.; Amin Nordin, S. Elevated interleukin-25 and its association to Th2 cytokines in systemic lupus erythematosus with lupus nephritis. *PloS one* **2019**, *14*, e0224707.
79. Singh, S.; Wu, T.; Xie, C.; Vanarsa, K.; Han, J.; Mahajan, T.; Oei, H.B.; Ahn, C.; Zhou, X.J.; Putterman, C.; et al. Urine VCAM-1 as a marker of renal pathology activity index in lupus nephritis. *Arthritis research & therapy* **2012**, *14*, R164.
80. Sjowall, C.; Bentow, C.; Aure, M.A.; Mahler, M. Two-Parametric Immunological Score Development for Assessing Renal Involvement and Disease Activity in Systemic Lupus Erythematosus. *Journal of immunology research* **2018**, *2018*, 1294680.
81. Smith, M.A.; Henault, J.; Karnell, J.L.; Parker, M.L.; Riggs, J.M.; Sinibaldi, D.; Taylor, D.K.; Ettinger, R.; Grant, E.P.; Sanjuan, M.A.; et al. SLE Plasma Profiling Identifies Unique Signatures of Lupus Nephritis and Discoid Lupus. *Scientific reports* **2019**, *9*, 14433.
82. Stanley, S.; Mok, C.C.; Vanarsa, K.; Habazi, D.; Li, J.; Pedroza, C.; Saxena, R.; Mohan, C. Identification of Low-Abundance Urinary Biomarkers in Lupus Nephritis Using Electrochemiluminescence Immunoassays. *Arthritis Rheumatol* **2019**, *71*, 744-755, doi:10.1002/art.40813.
83. Stanley, S.; Vanarsa, K.; Soliman, S.; Habazi, D.; Pedroza, C.; Gidley, G.; Zhang, T.; Mohan, S.; Der, E.; Suryawanshi, H.; et al. Comprehensive aptamer-based screening identifies a spectrum of urinary biomarkers of lupus nephritis across ethnicities. *Nature communications* **2020**, *11*, 2197.
84. Taha, H.A.; Abdallah, N.H.; Salem, M.N.; Hamouda, A.H.; Abd Elazeem, M.I.; Eesa, N.N. Urinary and tissue monocyte chemoattractant protein1 (MCP1) in lupus nephritis patients. *The Egyptian Rheumatologist* **2017**, *39*, 145-150, doi:https://doi.org/10.1016/j.ejr.2017.01.004.
85. Tang, C.; Fang, M.; Tan, G.; Zhang, S.; Yang, B.; Li, Y.; Zhang, T.; Saxena, R.; Mohan, C.; Wu, T. Discovery of Novel Circulating Immune Complexes in Lupus Nephritis Using Immunoproteomics. *Front Immunol* **2022**, *13*, 850015, doi:10.3389/fimmu.2022.850015.

86. Torres-Salido, M.T.; Sanchis, M.; Sole, C.; Moline, T.; Vidal, M.; Vidal, X.; Sola, A.; Hotter, G.; Ordi-Ros, J.; Cortes-Hernandez, J. Urinary Neuropilin-1: A Predictive Biomarker for Renal Outcome in Lupus Nephritis. *International journal of molecular sciences* **2019**, *20*.
87. Treamtrakanpon, W.; Tantivitayakul, P.; Benjachat, T.; Somparn, P.; Kittikowit, W.; Eiam-ong, S.; Leelahavanichkul, A.; Hirankarn, N.; Avihingsanon, Y. APRIL, a proliferation-inducing ligand, as a potential marker of lupus nephritis. *Arthritis research & therapy* **2012**, *14*, R252.
88. Urrego, T.; Ortiz-Reyes, B.; Vanegas-Garcia, A.L.; Munoz, C.H.; Gonzalez, L.A.; Vasquez, G.; Gomez-Puerta, J.A. Utility of urinary transferrin and ceruloplasmin in patients with systemic lupus erythematosus for differentiating patients with lupus nephritis. *Transferrina y ceruloplasmina en orina de pacientes con lupus eritematoso sistemico. Son utiles para diferenciar pacientes con nefritis lupica?* **2020**, *16*, 17-23.
89. Urrego-Callejas, T.; Álvarez, S.S.; Arias, L.F.; Reyes, B.O.; Vanegas-García, A.L.; González, L.A.; Muñoz-Vahos, C.H.; Vásquez, G.; Quintana, L.F.; Gómez-Puerta, J.A. Urinary levels of ceruloplasmin and monocyte chemoattractant protein-1 correlate with extra-capillary proliferation and chronic damage in patients with lupus nephritis. *Clin Rheumatol* **2021**, *40*, 1853-1859, doi:10.1007/s10067-020-05454-0.
90. Vanarsa, K.; Soomro, S.; Zhang, T.; Strachan, B.; Pedroza, C.; Nidhi, M.; Cicalese, P.; Gidley, C.; Dasari, S.; Mohan, S.; et al. Quantitative planar array screen of 1000 proteins uncovers novel urinary protein biomarkers of lupus nephritis. *Ann Rheum Dis* **2020**, *79*, 1349-1361, doi:10.1136/annrheumdis-2019-216312.
91. Vincent, F.B.; Kandane-Rathnayake, R.; Hoi, A.Y.; Slavin, L.; Godsell, J.D.; Kitching, A.R.; Harris, J.; Nelson, C.L.; Jenkins, A.J.; Chrysostomou, A.; et al. Urinary B-cell-activating factor of the tumour necrosis factor family (BAFF) in systemic lupus erythematosus. *Lupus* **2018**, *27*, 2029-2040.
92. Wang, Y.; Huang, X.; Cai, J.; Xie, L.; Wang, W.; Tang, S.; Yin, S.; Gao, X.; Zhang, J.; Zhao, J.; et al. Clinicopathologic Characteristics and Outcomes of Lupus Nephritis With Antineutrophil Cytoplasmic Antibody: A Retrospective Study. *Medicine* **2016**, *95*, e2580.
93. Wang, Y.; Tao, Y.; Liu, Y.; Zhao, Y.; Song, C.; Zhou, B.; Wang, T.; Gao, L.; Zhang, L.; Hu, H. Rapid detection of urinary soluble intercellular adhesion molecule-1 for determination of lupus nephritis activity. *Medicine* **2018**, *97*, e11287.
94. Wang, S.; Wu, M.; Chiriboga, L.; Zeck, B.; Belmont, H.M. Membrane attack complex (mac) deposition in lupus nephritis is associated with hypertension and poor clinical response to treatment. *Seminars in arthritis and rheumatism* **2018**, *48*, 256-262.
95. Wang, S.; Shang, J.; Xiao, J.; Zhao, Z. Clinicopathologic characteristics and outcomes of lupus nephritis with positive antineutrophil cytoplasmic antibody. *Renal failure* **2020**, *42*, 244-254.
96. Wantanasiri, P.; Satirapoj, B.; Charoenpitakchai, M.; Aramwit, P. Periostin: a novel tissue biomarker correlates with chronicity index and renal function in lupus nephritis patients. *Lupus* **2015**, *24*, 835-845.
97. Wolf, B.J.; Spainhour, J.C.; Arthur, J.M.; Janech, M.G.; Petri, M.; Oates, J.C. Development of Biomarker Models to Predict Outcomes in Lupus Nephritis. *Arthritis & rheumatology (Hoboken, N.J.)* **2016**, *68*, 1955-1963.
98. Wu, T.; Du, Y.; Han, J.; Singh, S.; Xie, C.; Guo, Y.; Zhou, X.J.; Ahn, C.; Saxena, R.; Mohan, C. Urinary angiostatin--a novel putative marker of renal pathology chronicity in lupus nephritis. *Molecular & cellular proteomics : MCP* **2013**, *12*, 1170-1179.
99. Wu, J.; Wei, L.; Wang, W.; Zhang, X.; Chen, L.; Lin, C. Diagnostic value of progranulin in patients with lupus nephritis and its correlation with disease activity. *Rheumatology international* **2016**, *36*, 759-767.
100. Wu, T.; Ding, H.; Han, J.; Arriens, C.; Wei, C.; Han, W.; Pedroza, C.; Jiang, S.; Anolik, J.; Petri, M.; et al. Antibody-Array-Based Proteomic Screening of Serum Markers in Systemic Lupus Erythematosus: A Discovery Study. *Journal of proteome research* **2016**, *15*, 2102-2114.
101. Wu, T.; Xie, C.; Han, J.; Ye, Y.; Singh, S.; Zhou, J.; Li, Y.; Ding, H.; Li, Q.-z.; Zhou, X.; et al. Insulin-Like Growth Factor Binding Protein-4 as a Marker of Chronic Lupus Nephritis. *PloS one* **2016**, *11*, e0151491.
102. Xia, Y.-R.; Li, Q.-R.; Wang, J.-P.; Guo, H.-S.; Bao, Y.-Q.; Mao, Y.-M.; Wu, J.; Pan, H.-F.; Ye, D.-Q. Diagnostic value of urinary monocyte chemoattractant protein-1 in evaluating the activity of lupus nephritis: a meta-analysis. *Lupus* **2020**, *29*, 599-606.
103. Yang, Z.; Zhang, Z.; Qin, B.; Wu, P.; Zhong, R.; Zhou, L.; Liang, Y. Human Epididymis Protein 4: A Novel Biomarker for Lupus Nephritis and Chronic Kidney Disease in Systemic Lupus Erythematosus. *Journal of clinical laboratory analysis* **2016**, *30*, 897-904.
104. Yap, D.Y.H.; Yung, S.; Zhang, Q.; Tang, C.; Chan, T.M. Serum level of proximal renal tubular epithelial cell-binding immunoglobulin G in patients with lupus nephritis. *Lupus* **2016**, *25*, 46-53.
105. Yu, K.Y.; Yung, S.; Chau, M.K.; Tang, C.S.; Yap, D.Y.; Tang, A.H.; Ying, S.K.; Lee, C.K.; Chan, T.M. Clinico-pathological associations of serum VCAM-1 and ICAM-1 levels in patients with lupus nephritis. *Lupus* **2021**, *30*, 1039-1050.
106. Zhang, T.; Li, H.; Vanarsa, K.; Gidley, G.; Mok, C.C.; Petri, M.; Saxena, R.; Mohan, C. Association of Urine sCD163 With Proliferative Lupus Nephritis, Fibrinoid Necrosis, Cellular Crescents and Intrarenal M2 Macrophages. *Frontiers in immunology* **2020**, *11*, 671.
107. Zhang, T.; Duran, V.; Vanarsa, K.; Mohan, C. Targeted urine proteomics in lupus nephritis - a meta-analysis. *Expert Rev Proteomics* **2020**, *17*, 767-776, doi:10.1080/14789450.2020.1874356.
